# The Influence of the 4-Diethylaminophenyl Substituent on the Physicochemical Properties of Phenanthro[9,10-*d*]imidazole Derivatives in the Context of Electroluminescent Applications

**DOI:** 10.3390/ma19010055

**Published:** 2025-12-23

**Authors:** Agnieszka Krawiec, Michał Filapek, Sławomir Kula

**Affiliations:** Institute of Chemistry, Faculty of Science and Technology, University of Silesia, Szkolna 9 St., 40-007 Katowice, Poland; agnieszka.krawiec@us.edu.pl (A.K.); michal.filapek@us.edu.pl (M.F.)

**Keywords:** imidazole, phenanthroimidazole derivatives, phenanthro[9,10-*d*]imidazole derivatives, electroluminescence, light emitting devices

## Abstract

Does position matter? In many respects, it certainly does, but does it also matter in the case of a functional group such as 4-diethylaminophenyl in the structure of phenanthro[9,10-*d*]imidazole derivatives? We attempt to answer this question in this article by considering selected physicochemical properties of the presented compounds. Therefore, in this work, four phenanthro[9,10-*d*]imidazole derivatives (**AM-0**–**AM-3**) were obtained by Debus-Radziszewski condensation. All derivatives were purified, and their structures were confirmed using NMR spectroscopy. The synthesized compounds were then compared for their thermal, electrochemical, and optical properties. This demonstrated that the derivatives (**AM-0** and **AM-1**) containing a 4-diethylaminophenyl substituent at the C2 position exhibit better physicochemical parameters than the other compounds, particularly in terms of thermal stability, energy gap, and even quantum yield. In the case of the latter parameter, derivatives containing 4-diethylaminophenyl at the C2 position show an increase of up to 15–30% (depending on the solvent used) compared to the compound containing the considered substituent at N1. The obtained research results were compared with DFT calculations to gain a deeper understanding of the experiments performed.

## 1. Introduction

Intensive technological development means that the requirements placed on chemical compounds used in practice are constantly increasing. This applies not only to the price and purity of the chemicals under consideration, but also, in particular, to the expected physicochemical properties. This trend is largely observed in organic electronics. This field of science and technology currently places particular requirements on chemical compounds in terms of their physicochemical properties. This often results from the fact that the better the physicochemical parameters of a chemical compound, the better the application for which it was designed. A prime example of this type is structural elements whose operation is based on the phenomenon of electroluminescence, such as organic light-emitting diodes (OLEDs) [1,2,3,4,5] and light-emitting electrochemical cells (LECs) [6,7,8,9,10]. To a large extent, the final parameters of OLEDs and LECs depend on the chemical compounds used in the individual layers, with particular emphasis on the emissive layer. In recent years, great hopes have been placed on emitters based on organic compounds. This is mainly due to the numerous advantages of such derivatives, including repeatable and efficient synthesis, a precisely defined structure that can be achieved using a variety of available methods, extensive processability thanks to excellent solubility in organic solvents, and the ability to modify physicochemical properties (at the synthesis stage) through the appropriate selection of substituents.

**Figure 1:**
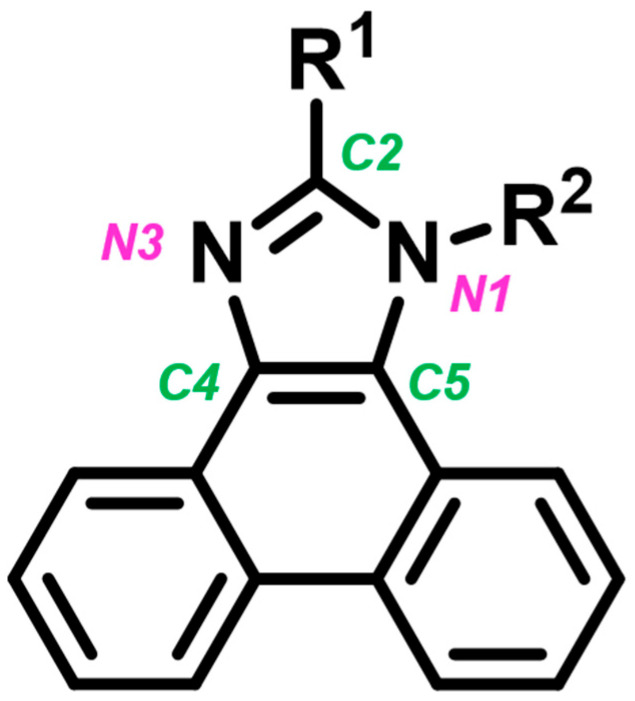
Structure of phenanthro[9,10-*d*]imidazole derivatives with numbering of the central imidazole ring, which is the core of the molecule.

An example of chemical compounds that have been intensively studied in recent years for use as emitters in OLEDs and LECs is phenanthro[9,10-*d*]imidazole derivatives [11,12,13,14,15,16,17,18,19,20,21,22,23,24,25,26,27,28,29,30,31,32,33]. In the case of these molecules, the heterocyclic imidazole ring core is connected to two substituents at the N1 and C2 positions, as well as three aromatic rings in the phenanthrene system (attached to the core through the C4 and C5 positions)—Figure 1. The appropriate selection of substituents enables the tailoring of the physicochemical properties of the compounds to meet the specific needs of various applications. As a result, many phenanthro[9,10-*d*]imidazole derivatives have been described in the literature, demonstrating high thermal stability and excellent electrochemical, optical, and electroluminescent properties, which are beneficial for use in OLEDs and LECs [11,12,13,14,15,16,17,18,19,20,21,22,23,24,25,26,27,28,29,30,31,32,33].

However, the vast majority of phenanthro[9,10-*d*]imidazole derivatives described in the literature differ in the substituent at the C2 position [11,12,13,14,15,16,17,18,19,20,21,22,23,24,25,26,27,28,29,30,31,32,33]. The indicated structural fragment is introduced into the structure of the final molecule via an aldehyde used as a substrate in the Debus-Radziszewski condensation reaction [15,16,18,19,20,22,26,28,29,32,33]. The wide selection of commercially available and synthetically advantageous aldehydes makes it easy to obtain a wide range of compounds, differentiated by their C2 position. In contrast, the N1 substituent is introduced by using an amine [15,16,18,19,20,22,26,28,29,32,33,34]. This is significantly more difficult, as the selection of commercially available and easily synthesized amines is considerably smaller than that of aldehydes. Furthermore, the reactivity of amines in the Debus-Radziszewski condensation reaction varies considerably. Therefore, the question arises: which of the considered positions (C2 and N1) will be more advantageous in terms of introducing a selected substituent or functional group that modifies physicochemical properties?

Therefore, in the research described in this article, we attempted to compare selected physicochemical properties of phenanthro[9,10-*d*]imidazole derivatives differing in the position of the 4-diethylaminophenyl substituent at the N1 and C2 positions (**AM-1** and **AM-2**). The results were enriched by a comparison with two phenanthro[9,10-*d*]imidazole derivatives serving as reference systems. The first is a derivative (**AM-0**) containing a 4-diethylaminophenyl substituent at C2 and an N-H moiety at the N1 position. The second is 1,2-diphenyl-1*H*-phenanthro[9,10-*d*]-imidazole (**AM-3**), a derivative containing phenyl substituents at the N1 and C2 positions. All compounds were compared for their thermal, electrochemical, and optical properties. The aim was to determine the effect of the 4-diethylaminophenyl substituent on the final physicochemical properties of phenanthro[9,10-*d*]imidazole derivatives. Also, it allowed us to determine which position, N1 or C2, of the 4-diethylaminophenyl group has a more favorable effect on the expected properties from the perspective of using phenanthro[9,10-*d*]imidazole derivatives in OLEDs and LECs.

## 2. Experimental Section

All details concerning the chemicals, materials, apparatus, experimental procedures and DFT calculations are provided in the Supporting Information (SI). The SI also includes NMR spectra, TGA thermograms, voltammograms, absorption and emission spectra of the compounds.

## 3. Results and Discussion

### 3.1. Synthesis

**Figure 2:**
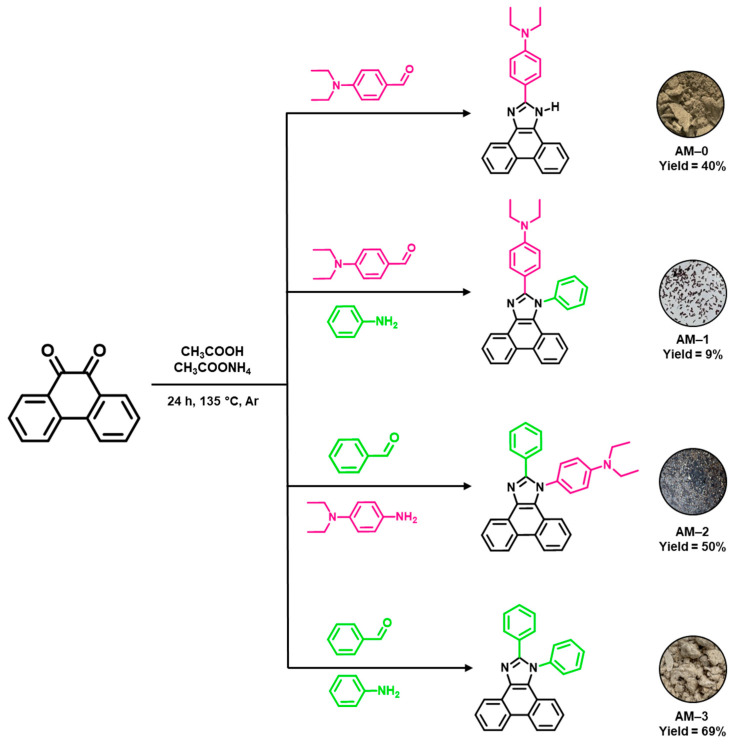
Synthesis of compounds **AM-0**–**AM-3** with yields and photographs of the powders.

All syntheses were performed based on the one-step Debus-Radziszewski condensation reaction [15,16,18,19,20,22,26,28,29,32,33]. In this method, mixing a diketone, an aldehyde, and an amine in an acidic medium leads to the formation of an imidazole derivative. The aldehyde introduces a substituent at the C2 position into the molecular structure. A suitably selected amine, in turn, provides the N1 substituent. If only the diketone, aldehyde, and ammonium acetate are used in the reaction, a hydrogen atom (N-H group) will be present at the N1 position of the resulting imidazole. In this work, four phenanthro[9,10-*d*]imidazole derivatives (**AM-0**, **AM-1**, **AM-2**, **AM-3**) were obtained, differing in the substituents at the C2 and N1 positions. For this purpose, 9,10-phenanthroquinone, the appropriate aldehyde (benzaldehyde or 4-diethylaminobenzaldehyde), amine (aniline or *N*,*N*-diethyl-p-phenylenediamine), and ammonium acetate were used in the reaction (Figure 2). The reaction medium was acetic acid. The syntheses were carried out at the boiling point of the reaction mixtures, under an argon atmosphere. After 24 h, the obtained precipitates were precipitated in water and filtered on filter paper. The residual ammonium acetate and acetic acid were removed by washing the precipitates with distilled water. To purify compounds **AM-1**, **AM-2**, and **AM-3**, column chromatography and crystallization in methanol were performed. Compound **AM-0** was purified by double crystallization in methanol. As a result, four phenanthro[9,10-*d*]imidazole derivatives were obtained in yields ranging from 9 to 69% (Figure 2). The structures of the obtained compounds were confirmed by NMR spectroscopy (^1^H and ^13^C). Detailed experimental procedures and NMR spectra are provided in the Supporting Information (Sections S3 and S4).

### 3.2. Thermal Properties

To investigate the thermal properties of the obtained molecules, thermogravimetric analysis (TGA) was performed and melting points were measured. Based on the conducted studies, 5% and 10% mass loss (T_5_, T_10_), decomposition temperatures (T_max_), and melting points of the tested compounds (T_m_) were determined—Table 1. Thermograms of the tested compounds are provided in the Supporting Information (Figure S1). Of the analyzed compounds, the **AM-0** derivative exhibits the highest thermal stability, as evidenced by a 5% mass loss occurring only at 312 °C. Comparison of the **AM-0** and **AM-1** derivatives, which share the same structural fragment, 4-diethylaminophenyl at the C2 position, enables an assessment of the effect of the substituent at the N1 position. Introduction of a phenyl group at N1 (**AM-1**) results in a decrease in thermal stability—a 5% mass loss is observed already at 289 °C. Compounds **AM-2** and **AM-3**, which contain a phenyl group at the C2 position, enable a similar comparison of the effect of substituents at the N1 position. In this case, the **AM-2** derivative with the 4-diethylaminophenyl group exhibits higher stability. For **AM-3** (with the phenyl group at the N1 position), a 5% mass loss occurs at 257 °C, making it the least stable compound among the derivatives studied. Based on the analysis, it can be concluded that the presence of the 4-diethylaminophenyl group at both the C2 and N1 positions promotes increased thermal stability of the compounds. Notably, the substituent at the C2 position has a greater impact on thermal stability—the 4-diethylaminophenyl substituent (**AM-1**, T_5_ = 289 °C) at this position provides higher stability than the phenyl substituent (**AM-2**, T_5_ = 283 °C). For all derivatives, complete decomposition occurs above 300 °C—the highest T_max_ is observed for **AM-2** (379 °C) and the lowest for **AM-3** (347 °C). Melting was also observed for each compound in the range of 186–312 °C. The melting point decreased in the series **AM-0** > **AM-1** > **AM-3** > **AM-2**.

### 3.3. Redox Behavior

The next stage of the research was a series of measurements using the differential pulse voltammetry (DPV) and cyclic voltammetry (CV) method which were used to study the electrochemical properties of dissolved substances. DPV is a sensitive technique in which a series of voltage pulses is superimposed on potential stair steps. Thus, only the faradaic current is examined, which allows for accurate redox analysis of the tested samples. CV measures the electric current flowing through the electrochemical system in response to a linearly changing potential of the working electrode, which in turn enables the study of the thermodynamics of electrode processes. These measurements allow the determination of ionization potential (IP), electron affinity (EA), and energy gap (Eg) of the investigated compounds (assuming the IP of ferrocene equals −5.1 eV [34]. This, finally, allows us to calculate the energy band gap (Eg), which is the key value. Representative voltammograms showing the redox behavior of the compounds are shown below. All of the reduction and oxidation voltammograms obtained during the research were placed in SI (Figure S2). Additionally, a summary table detailing the electrochemical properties is provided below (Table 2).

The first part of the electrochemical studies began with the analysis of the redox properties of the molecules using the DPV method. All derivatives underwent the oxidation process at relatively low potentials—i.e., below 1V—confirming the high electron density within the molecules and their potential for being p-doped. The precise E_ox_ values varied significantly depending on the type of substituents, both at the C2 and N1 positions. This indicates that oxidation is localized to (or involves a significant contribution from) the imidazole moiety. The lowest E_ox_ value was measured for the **AM-0** derivative (i.e., 0.08 V), which contains an N-H bond. This bond is highly polarized—the hydrogen is somewhat acidic (as confirmed by ^1^H NMR analysis). Therefore, the electron density is shifted to the imidazole ring, stabilizing the oxidized forms. Bearing this in mind, it is easy to predict that the **AM-3** derivative undergoes oxidation at the highest potential—it has two phenyl substituents (both at the N1 and C2 positions), which have the weakest electron-donating properties. In contrast, the **AM-1** and **AM-2** derivatives (having a phenyl substituent in one position and a p-aniline substituent in the other), resulting in oxidation rates intermediate between those observed for **AM-0** and **AM-3**. However, it is worth noting that substitution at the C2 position has a more favorable effect on the stabilization of oxidized forms than substitution at the N1 position.

**Figure 3:**
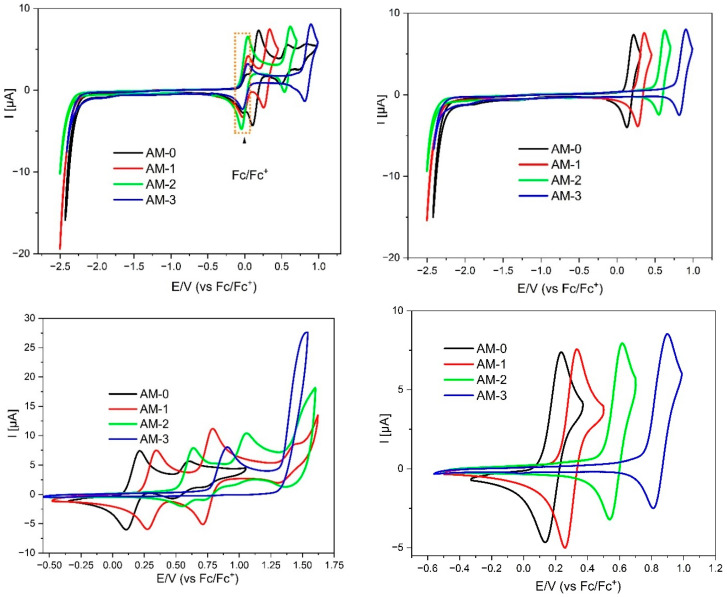
Cyclic voltammograms of the investigated compounds with sweep rate ν = 100 mV/s, 0.1 M Bu_4_NPF_6_ in CH_2_Cl_2_.

Most importantly, the first oxidation state for this group of compounds is fully thermodynamically reversible (Figure 3). Additionally, (except for the **AM-3** derivative), the second oxidation state is also reversible. This demonstrates excellent p-doping properties, as well as the fact that the molecules can, to some extent, act as hole conductors, for example, in an LEC device.

**Figure 4:**
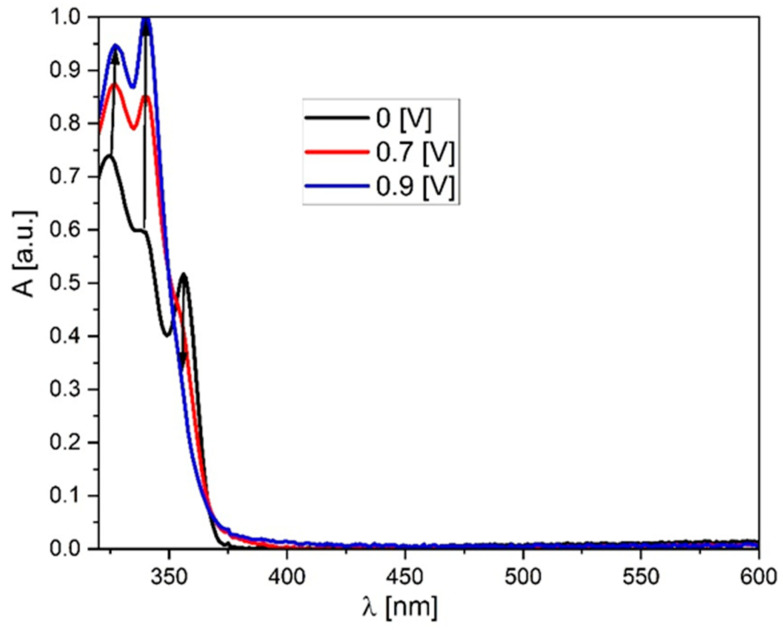
UV-Vis spectroelectrochemistry of the **AM-0** derivative in DCM solution (c = 1 × 10^−4^ mol/L, as an inset on the graph potentials vs. Fc/Fc + redox couple).

In turn, analyzing the properties of the molecules during reduction, they behave quite similarly (all of them were reducing below −2.25 V), which is typical for the phenantorimidazole core [35]. Taking both these values (E_ox_ and E_red_) into account, the electrochemical energy gap can be calculated—it lies between 2.33 eV (**AM-0**) and 3.11 eV (**AM-3**). This value is slightly lower than the energy gap determined by UV-VIS absorption spectroscopy, indicating that the HOMOs and LUMOs are (at least partially) separated in space. Spectroelectrochemical measurements were also performed for the studied compounds. As can be seen in Figure 4, the appearance of the absorption band changes after oxidation. Initially, three local minima can be distinguished at 325, 339, and 356 nm. However, after oxidation, the lowest energy band disappears, and the other bands increase, with the middle band becoming the most intense. What is crucial, however, is that after dedoping (i.e., the molecule returns to its neutral form), the spectrum returns to its original shape, further demonstrating the high stability and reversibility of oxidation. It is also worth noting that the absorption region remains unchanged—oxidation does not produce additional bands that could negatively impact the performance of OLED or LED devices built using this group of compounds.

### 3.4. DFT Calculations

To better understand how the position of substitution affects the properties of the presented compounds, DFT calculations were performed. Geometry optimization of the **AM-0–AM-3** derivatives was performed using density functional theory (DFT) calculations at the PBE0/def2TZVP level, implemented in the Gaussian 16 software package. Transition energies and oscillator strengths of the compounds were calculated using a time-dependent DFT (TD-DFT) approach.

Looking at the structures of the presented compounds, one might get the impression that they are similar. **AM-0** and **AM-1** have the same substituent (4-diethylaminophenyl) at the C2 position. However, they differ in the substitution at the N1 position. Analyzing the dihedral angles between the 1*H*-phenanthro[9,10-*d*]imidazole plane and the substituent at the C2 position, a significant difference can be observed between these compounds. **AM-0** exhibits a practically planar conformation (Figure S3), which may result in substantial fluorescence quenching at high compound concentrations, e.g., in pure layers [36]. **AM-1**, having a phenyl group at the N1 position, is not planar. This compound exhibits a distorted molecular geometry due to two torsion angles between the core plane and the C2 substituent (33°) and the phenyl ring at the N1 position (77°) (Figure S3). The twisted geometry of the compound may prevent the molecules from tightly packing at increased concentrations, resulting in suppressed fluorescence quenching in pure layers [36,37]. However, when we compare **AM-1** to compound **AM-3**, we observe a certain similarity between them. **AM-3** also does not have a planar conformation, and the dihedral angle between the phenyl group in N1 and the molecular core is the same. Changing the substituent at the C2 position does not affect this angle. Interestingly, the phenyl substituent attached at the C2 position increases the torsion angle (between C2 and the core) in the final structure of the compound compared to the 4-diethylaminophenyl fragment. Considering **AM-2**, the substituent reciprocal of **AM-1**, one can observe an increase in the dihedral angle between the N1 planes and the core compared to **AM-1**. Inserting the 4-diethylaminophenyl substituent at the N1 position decreases the torsion angle between C2 and the core compared to **AM-3** (Figure S3). In the case of **AM-1**, **AM-2**, and **AM-3**, the core of the molecule is relatively well coupled to the substituents at the C2 position, which lie on the same axis, but due to the large angles, it exhibits weaker electronic communication with the pendant units at the N1 position. Analysis of the highest occupied molecular orbital (HOMO) showed that for **AM-0**, **AM-1**, and **AM-3**, this orbital was distributed throughout the molecule (except the highly twisted phenyl moiety attached to the N1 position in **AM-1** and **AM-3**). The 1*H*-phenanthro[9,10-*d*]imidazole core of **AM-1** and **AM-3** is well coupled to the substituents attached at the C2 position, which lie on the same axis. However, it exhibits weak electronic communication with the pendant unit at the N1 position. The HOMO of **AM-2** was dominated primarily by the orbitals of the 4-diethylaminophenyl moiety located at the N1 position. Moreover, the HOMO also encompasses orbitals originating from the core and the phenyl moiety on the same axis. In the case of this molecule, the strong torsion angle did not constitute a barrier to electron flow. As can be seen in Figure S4, the lowest unoccupied molecular orbital (LUMO) for **AM-2** and **AM-3** encompasses the entire molecule, except for the motifs at the N1 position. Completely opposite behavior was exhibited by molecules possessing a substituent with pronounced electron-donating properties (**AM-0** and **AM-1**). These compounds were characterized by a shift in the LUMO toward the 1*H*-phenanthro[9,10-*d*]imidazole core. Literature reports confirmed this behavior [36,37,38,39,40,41,42]. Electron affinity and ionization potential were calculated for all derivatives (Table 3). These values are close to the energies of the frontier orbitals. Compounds with an electron-rich substituent at the C2 position destabilized the HOMO the most. Also, they exhibited the greatest ability to undergo reduction processes (Table 3). The energy gap, calculated from the difference between the IP and EA values, ranged from 3.56 eV to 3.90 eV. The trend of the energy gap results is consistent with the energy gap obtained from electrochemical measurements.

**Figure 5:**
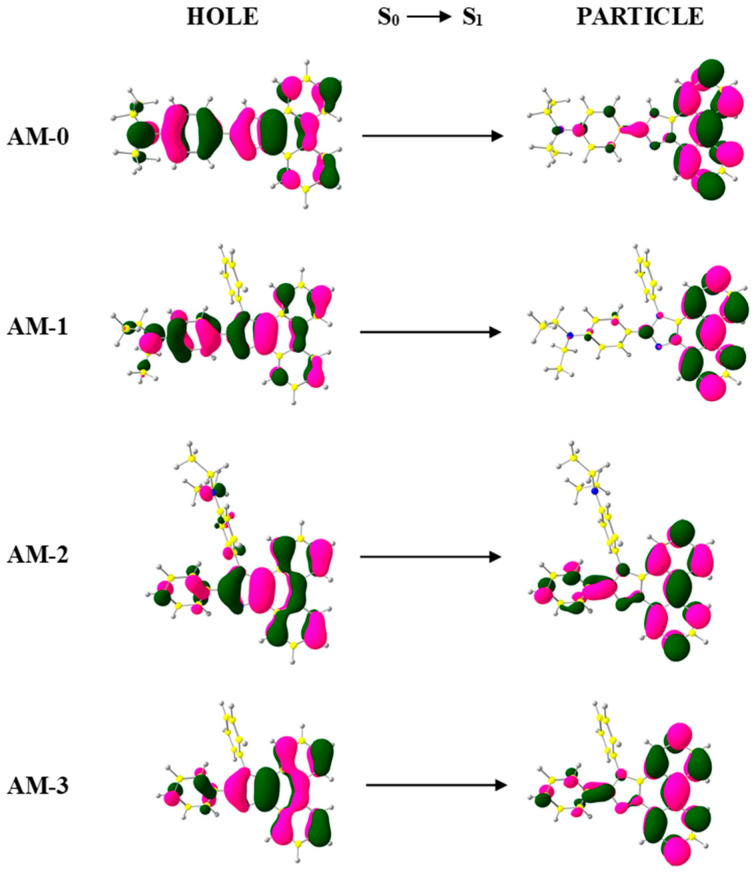
The S_0_→S_1_ transition NTO of **AM-0–AM-3**.

Furthermore, excited-state properties were calculated using TD-DFT (Figure S5 and Table S1). Analysis of natural transition orbitals (NTO) showed that the S_0_→S_1_ transition for **AM-3** exhibits predominantly locally excited (LE) character due to the completely overlapping “holes” and “particles” (Figure 5). Interestingly, for **AM-2**, the “hole” wave functions encompassed the entire molecule, while the “particles” were located on the main axis of the compound, giving the **AM-2** transitions an LE character. For **AM-0** and **AM-1**, this transition may have an HLCT character, as partial orbital separation is observed between the “hole” and “particle” wave functions (Figure 5) [38,39]. The optical gaps determined from TD-DFT calculations were in the range of 3.15–3.50 eV, which corresponded to the actual results.

### 3.5. Optical Properties

The optical absorption and fluorescence properties of compounds **AM-0–AM-3** were assessed by UV/Vis absorption and photoluminescence (PL) spectroscopy in dilute solutions (at a concentration of 2.5 × 10^−5^ mol/L) of six solvents with different dielectric constants. The obtained spectra are presented in the Supplementary Materials (Figures S6 and S7), and the detailed photophysical data are summarized in Table 4.

The properties of the presented derivatives **AM-0** to **AM-3**, observed in dilute solutions at low concentrations of the compounds, allowed for the revelation of the influence of intramolecular mechanisms. The recorded spectra for **AM-2** and **AM-3** exhibited similar absorption profiles, with the lowest energy band peak having a maximum value of approximately 360 nm (Figure S7). The bands in this range can be assigned to π − π* transitions originating from the 1*H*-phenanthro[9,10-*d*]imidazole core [43]. It is worth emphasizing that changing the substituent at the N1 position did not significantly affect the shape or the maximum absorption value. Compounds **AM-0** and **AM-1** with the 4-diethylaminophenyl substituent showed a spectrum with a clearly broadened lowest energy absorption band. This may indicate electron flow from the donor to the acceptor in these molecules. Furthermore, for **AM-0** and **AM-1**, an increase in the molar absorption coefficient was observed, which is typical for D-A molecules and results from an increased transition dipole moment [36]. In terms of absorption studies, no solvatochromic effect was observed. The emission curves for compounds **AM-2** and **AM-3** show minor differences in shape and position. The emission maxima with strong violet photoluminescence (PL) are in the range of 365–390 nm. The excitation character of both derivatives can be assigned to a locally excited state [43]. This is indicated by the fine structure of the spectra of both compounds (Figure S6). **AM-2** and **AM-3** do not exhibit a solvatochromic effect upon excitation. Interestingly, in this case, the N1 position did not significantly influence the position of the band maxima. For **AM-2** with a 4-diethylaminophenyl substituent at the N1 position, a distortion of the vibronic structure was observed in solvents with a higher dielectric constant. **AM-0** and **AM-1** are significantly bathochromically shifted upon excitation relative to **AM-2** and **AM-3** (Figure S7). Also, their emission spectra showed deep blue emission with a maximum above 400 nm. Solvent polarity in the case of **AM-0** and **AM-1** significantly affects the excited emission states. With increasing solvent polarity (toluene → DMSO), a red shift was observed. Initially, emission with a vibrational band structure was observed in toluene. This indicates that the excited states of these molecules in a nonpolar solvent are LE states [42]. With increasing solvent polarity, the double-peak emission gradually decreased and finally turned into single-peak emission (Figure S6). This may indicate a gradual transition to the HLCT state. The single-peak emission occurring in high-dielectric solvents may be caused by the influence of intramolecular charge distribution and intermolecular interactions by solvent polarity [41]. Fluorescence quantum yields (QY) for **AM-0** and **AM-1** ranged from 0.20 to 0.41. Such high quantum yields may be due to the HLCT nature of these molecules. As is well known, compounds exhibiting this characteristic benefit from both LE and CT processes. Moreover, the **AM-1** derivative with a phenyl substituent at the N1 position showed higher quantum yields than **AM-0**. This may be due to the inability of the substituent to form hydrogen bonds with the imidazole ring and the less rigid structure of this compound (Figure S3). Compounds **AM-2** and **AM-3** showed lower QY than **AM-0** and **AM-1**. Of the entire group, **AM-2** was characterized by the lowest QY values (Table 4). This is most likely due to the presence of the 4-diethylaminophenyl group at the N1 position. The structure of this compound is unfavorable in terms of charge transfer (the HOMO is located on the N1 substituent, and the LUMO is located on the core of the molecule).

## 4. Conclusions

The primary goal of this study was to determine the effect of the 4-diethylaminophenyl substituent at the N1 and C2 positions on selected physicochemical properties of phenanthro[9,10-*d*]imidazole derivatives. Therefore, four compounds (**AM-0**–**AM-3**) were obtained, differing in terms of the substituents at the considered positions. Analyzing the thermal properties of the studied derivatives, we observe that the 4-diethylaminophenyl group has a favorable effect on thermal stability compared to the phenyl substituents. Derivatives with the 4-diethylaminophenyl substituent at the C2 position (**AM-0**–**AM-1**) exhibit better thermal parameters, particularly when the N1 position contains only an N-H moiety (**AM-0**). Electrochemical properties of the studied compounds revealed that substitution of phenanthro[9,10-*d*]imidazole at the C2 position with a 4-diethylaminophenyl group had a more favorable effect on the stabilization of oxidized forms than substitution with a phenyl group. Additionally, the choice of the substituent at the N1 position also plays a role during the oxidation process. **AM-0** with an N-H motif at the N1 position underwent this process most readily. Absorption and emission properties were strongly related to the substituents at the C2 position. Their absorption profiles and maxima were similar in the **AM-2**–**AM-3** and **AM-0**–**AM-1** pairs. It is worth emphasizing that changing the substituent at the N1 position did not significantly affect the shape or maximum absorption value. Emission studies showed that compounds with a 4-diethylaminophenyl substituent at the C2 position were more red-shifted. Furthermore, these compounds exhibited solvatochromism. Substitution at the N1 position did not affect the emission maxima values. The modification of the N1 group in the described compounds affected the QY values. Considering the same emission character in the **AM-0**/**AM-1** and **AM-2**/**AM-3** pairs, the N1 substituent played a role in stiffening the structure or withdrawing electrons, which resulted in an increase or decrease in QY. To sum up, a detailed analysis of the physicochemical properties of the tested molecules (AM-0–AM-3) with particular emphasis on parameters such as thermal stability, energy gap and quantum efficiency showed that the suitability of the considered compounds for further application studies in OLEDs is classified as follows: AM-1 > AM-0 > AM-3 > AM-2.

## Figures and Tables

**Table 1:** Thermal parameters of the tested compounds.

Compound	T_5_ ^a^ [°C]	T_10_ ^b^ [°C]	T_max_ ^c^ [°C]	T_m_ ^d^ [°C]
**AM-0**	312	320	363	312
**AM-1**	289	310	368	220
**AM-2**	283	313	379	186
**AM-3**	257	285	347	202

^a^ T_5_—temperature of 5% mass loss, ^b^ T_10_—temperature of 10% mass loss, ^c^ T_max_—temperature of maximum decomposition rate, ^d^ T_m_—melting point.

**Table 2:** Data obtained from electrochemical measurements.

Compound	E_1red_ [V]	E_1ox_ [V]	E_2ox_ [V]	E_3ox_ [V]	IP ^1^ (eV)	EA ^2^ (eV)	Eg_(CV)_ ^3^ [eV]
**AM-0**	−2.25	0.08	0.52	-	−5.18	−2.85	2.33
**AM-1**	−2.25	0.23	-	-	−5.33	−2.85	2.48
**AM-2**	−2.25	0.50	0.96	1.37	−5.60	−2.85	2.75
**AM-3**	−2.35	0.76	1.33	-	−5.86	−2.75	3.11

^1^ Calculated from CV measurements: IP = −5.1 − E_ox_; ^2^ Calculated from CV measurements: EA = −5.1 − E_red_; ^3^ Eg_(CV)_ = E_ox_ (onset) − E_red_ (onset).

**Table 3:** Data obtained from DFT calculations.

Compound	HOMO_(DFT)_ [eV]	LUMO_(DFT)_ [eV]	IP_(DFT)_ [eV]	EA_(DFT)_ [eV]	Eg_(DFT)_ [eV]	IP—EA_(DFT)_ [eV]	Eg _(opt DFT)_ [eV]
**AM-0**	−5.29	−1.19	5.05	1.49	4.10	3.56	3.15
**AM-1**	−5.34	−1.18	5.05	1.47	4.16	3.58	3.25
**AM-2**	−5.77	−1.28	5.39	1.66	4.49	3.73	3.46
**AM-3**	−5.90	−1.33	5.62	1.72	4.58	3.90	3.50

**Table 4:** Optical properties of **AM-0–AM-3** derivatives.

Compound	Solvent	ε [10^4^ dm^3^·mol^−1^·cm^−1^]	λ_abs_ [nm]	λ_em_ [nm]	Stokes Shift * [nm]	QY	Eg_opt_
**AM-0**	toluene	4.18, 5.80, 3.12sh	289, 348, 380sh	401, 416	53	0.28	-
	CHCl_3_	2.01, 3.00, 1.97sh	317, 351, 378sh	418, 435	67	0.22	3.15
	THF	6.17, 5.50, 3.61, 5.31, 2.68sh	248, 262, 287, 347, 377sh	420	73	0.27	-
	MeCN	6.42, 5.24, 3.04, 5.42, 3.30sh	248, 263, 286, 344, 374sh	444	100	0.20	-
	DMSO	5.24, 3.18, 5.41, 3.29sh	264, 289, 351, 379sh	453	102	0.26	-
	MeOH	6.85, 5.73, 3.06, 5.40, 3.40	247, 260, 286, 342, 374	425	51	0.26	-
**AM-1**	toluene	2.64, 3.28, 1.58sh	290, 342, 375sh	398, 415	56	0.34	-
	CHCl_3_	1.67, 1.80, 2.35, 1.04sh	280, 289, 341, 375sh	429, 439	88	0.41	3.15
	THF	6.87, 3.56, 4.78, 2.46sh	250, 289, 342, 375sh	415	73	0.34	-
	MeCN	7.10, 3.28, 4.46, 2.23sh	251, 288, 336, 372sh	441	105	0.21	-
	DMSO	5.69, 3.23, 4.79, 2.66sh	261, 288, 344, 380sh	448	104	0.30	-
	MeOH	7.88, 3.42, 4.60, 2.41sh	250, 285, 330, 364sh	419	89	0.31	-
**AM-2**	toluene	3.79, 2.75, 1.12, 0.99	283, 312, 347, 364	373, 392, 413	9	0.20	-
	CHCl_3_	2.97, 4.80, 2.73, 0.94, 0.83	272, 278, 310, 347, 362	376, 392, 417	14	0.07	3.33
	THF	7.67, 6.30, 2.80, 1.05, 0.93	263, 272, 312, 346, 363	375, 389, 413	12	0.05	-
	MeCN	8.21, 6.41, 2.81, 0.86, 0.80	259, 272, 309, 345, 361	367, 384, 407	6	0.02	-
	DMSO	8.08, 6.45, 2.81, 0.96, 0.83	264, 273, 311, 347, 363	370, 387, 410	7	0.03	-
	MeOH	9.00, 5.91, 2.86, 0.85, 0.73	258, 273, 304, 339, 355	365, 383, 402	10	0.04	-
**AM-3**	toluene	2.00, 1.71, 1.58, 0.60, 0.52	278, 287, 310, 344, 360	372, 389, 412	12	0.21	-
	CHCl_3_	1.54, 1.48, 0.71, 0.60	284, 314, 345, 362	372, 391, 412	10	0.15	3.34
	THF	5.45, 1.67, 1.70, 0.73, 0.65	262, 287, 313, 344, 361	372, 389, 411	11	0.24	-
	MeCN	5.32, 1.53, 1.47, 0.50, 0.48	258, 285, 308, 342, 359	376, 389, 414	17	0.19	-
	DMSO	5.34, 1.64, 1.58, 0.59, 0.52	263, 287, 312, 344, 361	378, 390, 414	17	0.26	-
	MeOH	6.25, 1.71, 1.55, 0.50, 0.45	258, 284, 303, 338, 354	370, 384, 406	16	0.17	-

* λ_em_ − λ_abs_.

## Data Availability

The original contributions presented in this study are included in the article/Supplementary Materials. Further inquiries can be directed to the corresponding author.

## Supplementary Materials

The following supporting information can be downloaded at: https://www.mdpi.com/article/10.3390/ma19010055/s1. Figure S1. TGA thermogram—thermal properties of **AM-0–AM-3** series. Figure S2. Differential pulse voltammetry and cyclic voltammograms of **AM-0–AM-3** with sweep rate ν = 100 mV/s, 0.1 M Bu_4_NPF_6_ in CH_2_Cl_2_. Figure S3. Dihedral angle between the C2 or N1 substituent and the central core of 1H-phenanthro[9,10-*d*]imidazole for **AM-0**–**AM-3**. Figure S4. Contours of HOMO and LUMO orbitals for **AM-0**–**AM-3.** Figure S5. Experimental (pink line) absorption spectra and calculated transitions (black sticks) of **AM-0**–**AM-3** in dichloromethane. Table S1. Electronic transition energies assigned to the lowest absorption bands of the **AM-0**–**AM-3** wavelengths. Figure S6. Absorption and emission spectra in different solvents for **AM-0**–**AM-3.** Figure S7. Comparison of absorption and emission spectra for **AM-0**–**AM-3**.
