# Supplementary material for: The Influence of the 4-Diethylaminophenyl Substituent on the Physicochemical Properties of Phenanthro[9,10-d]imidazole Derivatives in the Context of Electroluminescent Applications
Source: Materials (Basel). 2025 Dec 23;19(1):55. doi: 10.3390/ma19010055 (PMC12786527; doi:10.3390/ma19010055)
Supplement: Supplementary file 1 [file materials-19-00055-s001.zip › materials-4019528-supplementary.pdf]

Supporting Information

The Influence of the 4-Diethylaminophenyl Substituent on the Physicochemical Properties of Phenanthro[9,10-*d*]imidazole Derivatives in the Context of Electroluminescent Applications

Agnieszka Krawiec, Michał Filapek and Sławomir Kula *

Institute of Chemistry, Faculty of Science and Technology, University of Silesia, Szkolna 9 St., 40-007 Katowice, Poland; agnieszka.krawiec@us.edu.pl (A.K.); michal.filapek@us.edu.pl (M.F.)

* Correspondence: slawomir.kula@us.edu.pl

1. **Materials** 2
2. **General methods – measurements** 2
3. **Synthesis** 2
4. **^{1}H NMR and ^{13}C NMR spectra** 5
5. **Thermal properties** 9
6. **Electrochemical properties** 10
7. **DFT calculation** 11
8. **Optical properties** 17

1. Materials

9,10-Phenanthrenedione (≥99%, Sigma-Aldrich), ammonium acetate (puriss. p.a., ACS reagent, reag. Ph. Eur., ≥98%, Sigma-Aldrich), benzaldehyde (>99.5%, Chemat), 4-diethylaminobenzaldehyde (99%, Sigma Aldrich), *N,N*-diethyl-p-phenylenediamine (97%, Sigma Aldrich), aniline (99%, Sigma Aldrich), ethyl acetate (pure, Chempur), dichloromethane (pure, Chempur), acetonitrile (for HPLC-GC, ≥99.8% (GC), Sigma-Aldrich), dichloromethane (for HPLC, ≥99.8%, contains amylene as stabilizer, Sigma-Aldrich), chloroform (for HPLC, ≥99.8%, amylene stabilized, Sigma-Aldrich), methanol (MeOH, pure for analysis, Chempur), DMSO-d_6 (pure for analysis, Chempur), tetrahydrofuran (pure for analysis, Eurochem BGD), glacial acetic acid (99,5% pure p. a, Chempur), dimethyl sulfoxide-d_6 (DMSO-d6, 99.8 atom % D, Sigma-Aldrich). All reactions were carried out under argon atmosphere.

2. General methods – measurements

Bruker NMR spectra were recorded using a Bruker Avance 500 instrument with DMSO-d6 as the solvent. Differential scanning calorimetry (DSC) and thermogravimetric analysis (TGA) were performed on a TA-DSC-25 and a TA-TGA-55, respectively. UV/Vis spectra were measured using a Perkin Elmer UV-VIS Lambda Bio 40 spectrophotometer, while photoluminescence emission spectra were obtained with a Hitachi Fluorescence Spectrophotometer F-7100. Electrochemical measurements were conducted using an Eco Chemie Autolab PGSTAT128n potentiostat. A glassy carbon electrode (2 mm diameter) served as the working electrode, while a platinum coil and a silver wire were used as the auxiliary and reference electrodes, respectively. All potentials were referenced against ferrocene (Fc), which was employed as an internal standard. Cyclic and differential pulse voltammetry experiments were carried out in a standard one-compartment cell using dichloromethane (DCM) (Carlo Erba, HPLC grade) as the solvent, under an argon atmosphere. A 0.2 M solution of Bu_4NPF_6 (Aldrich, 99%) was used as the supporting electrolyte.

3. Synthesis

2-(4-diethylaminophenyl)-1*H*-phenanthro[9,10-*d*]-imidazole (AM-0)

A mixture of 4-diethylaminobenzaldehyde (1.06 g, 6.00 mmol), 9,10-phenanthrenedione (1,25 g, 6.00 mmol), ammonium acetate (7,75 g, 100.00 mmol) and acetic acid (45 mL) was heated at reflux under argon atmosphere. After 24 h, the solid was collected by filtration and washed with distilled water. Crude product was purified by double crystallization using methanol as

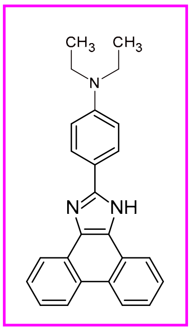

solvent. **AM-0** was obtained as brown solid with **40%** yield. **^{1}H NMR (500 MHz, DMSO-*d$_6$*) δ** 13.08 (s, 1H), 8.84 (d, J = 8.3 Hz, 2H), 8.57 – 8.53 (m, 2H), 8.11 (d, J = 8.9 Hz, 2H), 7.75 – 7.69 (m, 2H), 7.63 – 7.57 (m, 2H), 6.85 (d, J = 9.1 Hz, 2H), 3.44 (q, J = 7.0 Hz, 4H), 1.16 (t, J = 7.0 Hz, 6H). **^{13}C NMR (125 MHz, DMSO-*d$_6$*) δ** 150.85, 148.63, 128.11, 127.70, 127.38, 125.20, 124.31, 122.27, 117.34, 111.63, 44.17, 12.99.

2-(4-diethylaminophenyl)-1-phenyl-1*H*-phenanthro[9,10-*d*]-imidazole (AM-1)

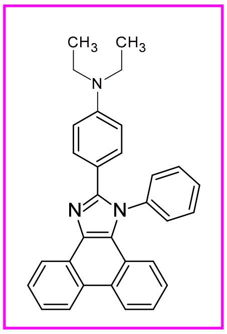

A mixture of 4-diethylaminobenzaldehyde (1.06 g, 6.00 mmol), aniline (2.79 g, 2.73 mL, 30.00 mmol), 9,10-phenanthrenedione (1,25 g, 6.00 mmol), ammonium acetate (7,75 g, 100.00 mmol) and acetic acid (45 mL) was heated at reflux under argon atmosphere. After 24 h, the solid was collected by filtration and washed with distilled water. The crude product was purified by column chromatography using silica gel and an eluent as a mixture of hexane : ethyl acetate (3:1). Then, the product was purified by crystallization using methanol as solvent. **AM-1** was obtained as dark brown solid with **50%** yield. **^{1}H NMR (500 MHz, DMSO-*d$_6$*) δ** 8.91 (d, J = 8.4 Hz, 1H), 8.86 (d, J = 8.7 Hz, 1H), 8.68 (dd, J = 7.8, 1.3 Hz, 1H), 7.80 – 7.63 (m, 7H), 7.55 – 7.48 (m, 1H), 7.42 – 7.36 (m, 2H), 7.34 – 7.27 (m, 1H), 7.03 (dd, J = 8.5, 1.3 Hz, 1H), 6.58 (d, J = 9.2 Hz, 2H), 3.36 (q, 4H), 1.08 (t, J = 7.0 Hz, 6H). **^{13}C NMR (125 MHz, DMSO-*d$_6$*) δ** 151.72, 148.19, 139.19, 130.91, 130.66, 130.58, 129.71, 128.57, 128.02, 127.80, 127.02, 126.04, 125.24, 124.93, 124.10, 122.97, 122.50, 120.41, 110.98, 44.01, 12.88.

2-phenyl-1-(4-diethylaminophenyl)-1*H*-phenanthro[9,10-*d*]-imidazole (AM-2)

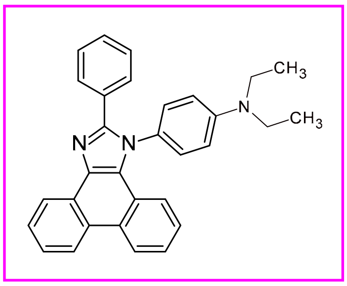

A mixture of benzaldehyde (0.64 g, 6.00 mmol), *N,N*-diethyl-p-phenylenediamine (3,12 g, 19.00 mmol) 9,10-phenanthrenedione (1,25 g, 6.00 mmol), ammonium acetate (7,75 g, 100.00 mmol) and acetic acid (45 mL) was heated at reflux under argon atmosphere. After 24 h, the solid was collected by filtration and washed with distilled water. Column chromatography on silica gel was performed to purify the crude product, using hexane : ethyl acetate in a 3:1 ratio as the eluent. Then, crude product was purified by crystallization using methanol as solvent. **AM-2** was obtained as dark brown solid with **9%** yield. **^{1}H NMR (500 MHz, DMSO-*d$_6$*) δ** 8.90 (d, J = 8.4 Hz, 1H), 8.86 (d, J = 8.3 Hz, 1H), 8.68 (dd, J = 7.9, 1.4 Hz, 1H), 7.79 – 7.72 (m, 1H), 7.70

– 7.62 (m, 3H), 7.58 – 7.51 (m, 1H), 7.41 – 7.29 (m, 7H), 6.84 (dd, J = 8.9 Hz, 2H), 3.43 (q, J = 7.0 Hz, 4H), 1.16 (t, J = 6.9 Hz, 6H). **^{13}C NMR (125 MHz, DMSO-*d_6*) δ** 151.42, 148.60, 136.69, 131.11, 129.97, 129.50, 129.27, 128.88, 128.63, 128.09, 127.82, 127.31, 127.08, 126.03, 125.52, 125.35, 124.82, 124.10, 123.31, 122.41, 120.87, 112.39, 44.19, 12.70.

2-phenyl-1-phenyl-1*H*-phenanthro[9,10-*d*]-imidazole (AM-3)

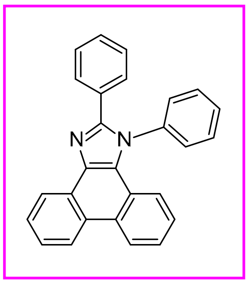

A mixture of benzaldehyde (0.64 g, 6.00 mmol), aniline (2.79 g, 2.73 mL, 30.00 mmol), 9,10-phenanthrenedione (1,25 g, 6.00 mmol), ammonium acetate (7,75 g, 100.00 mmol) and acetic acid (45 mL) was heated at reflux under argon atmosphere. After 24 hours, the resulting solid was isolated by filtration and rinsed with distilled water. The crude product was purified by column chromatography using silica gel and an eluent as a mixture of hexane : ethyl acetate (3:1). Then, the product was purified by crystallization using methanol as solvent. **AM-3** was obtained as light brown solid with **69%** yield. **^{1}H NMR (500 MHz, DMSO-*d_6*) δ** 8.94 (dd, J = 8.3, 1.0 Hz, 1H), 8.91 – 8.86 (m, 1H), 8.70 (dd, J = 7.8, 1.1 Hz, 1H), 7.82 – 7.75 (m, 1H), 7.74 – 7.67 (m, 6H), 7.62 – 7.53 (m, 3H), 7.42 – 7.31 (m, 4H), 7.09 (dd, J = 8.4, 1.3 Hz, 1H).**^{13}C NMR (125 MHz, DMSO-*d_6*) δ** 151.05, 138.65, 136.93, 130.79, 130.73, 130.69, 129.64, 129.61, 129.47, 128.98, 128.68, 128.24, 128.15, 127.95, 127.19, 127.11, 126.23, 125.69, 124.99, 124.16, 122.97, 122.49, 120.66.

4. ^{1}H NMR and ^{13}C NMR spectra

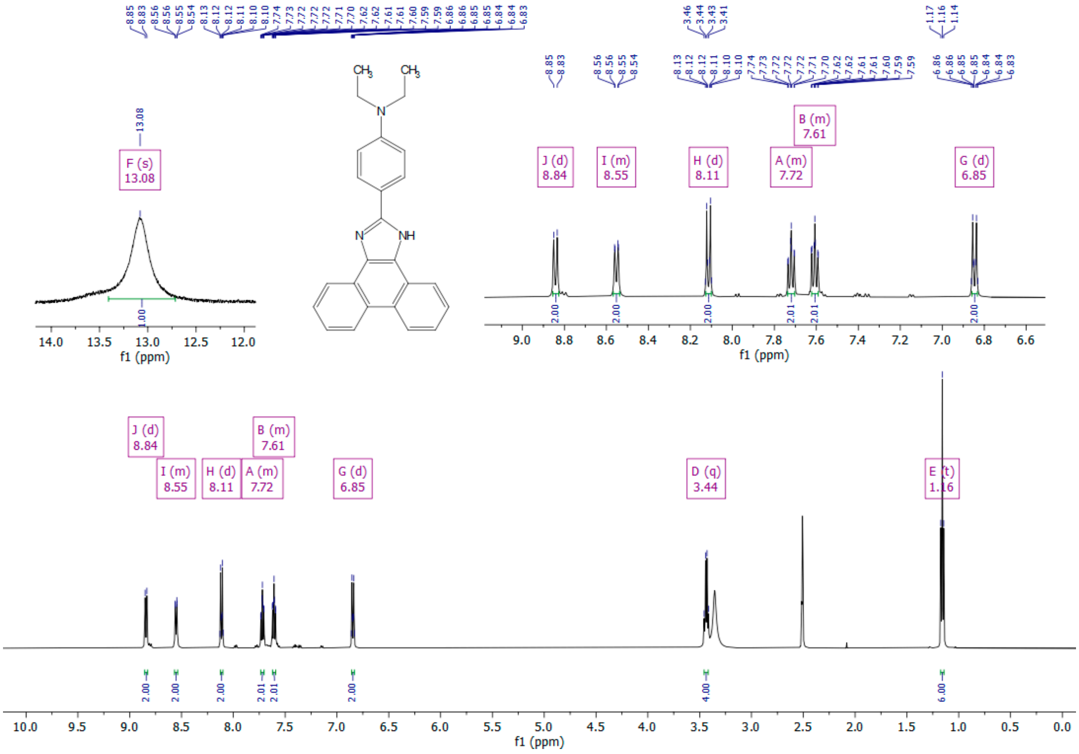

^{1}H NMR of AM-0

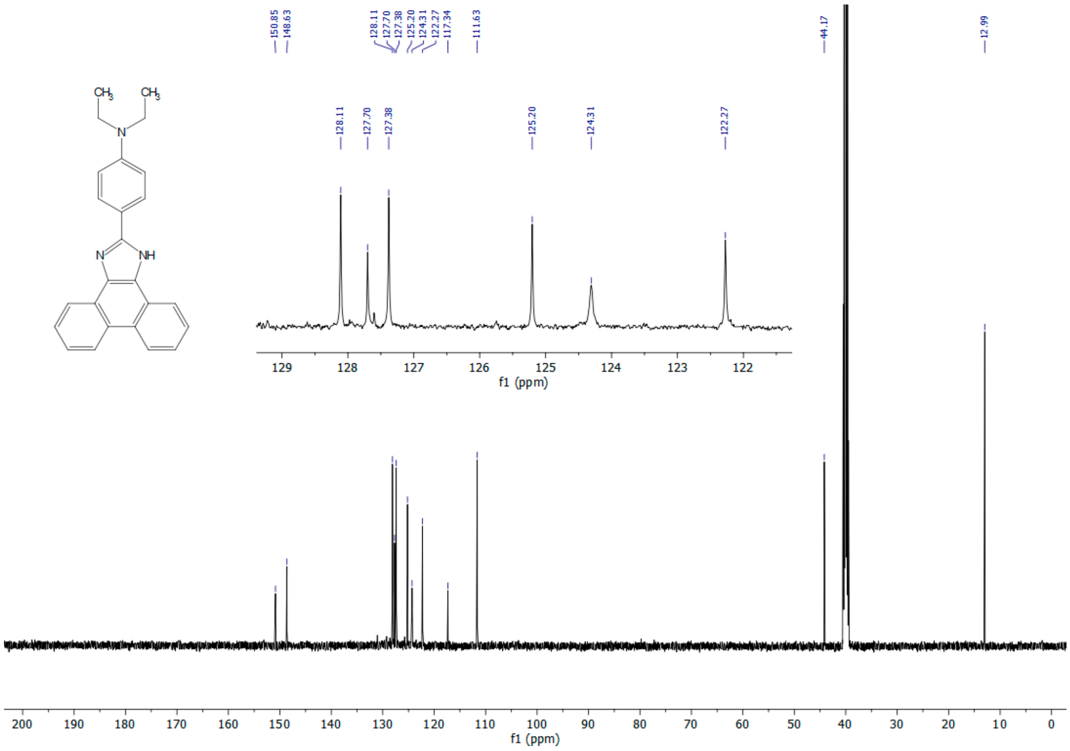

^{13}C NMR of AM-0

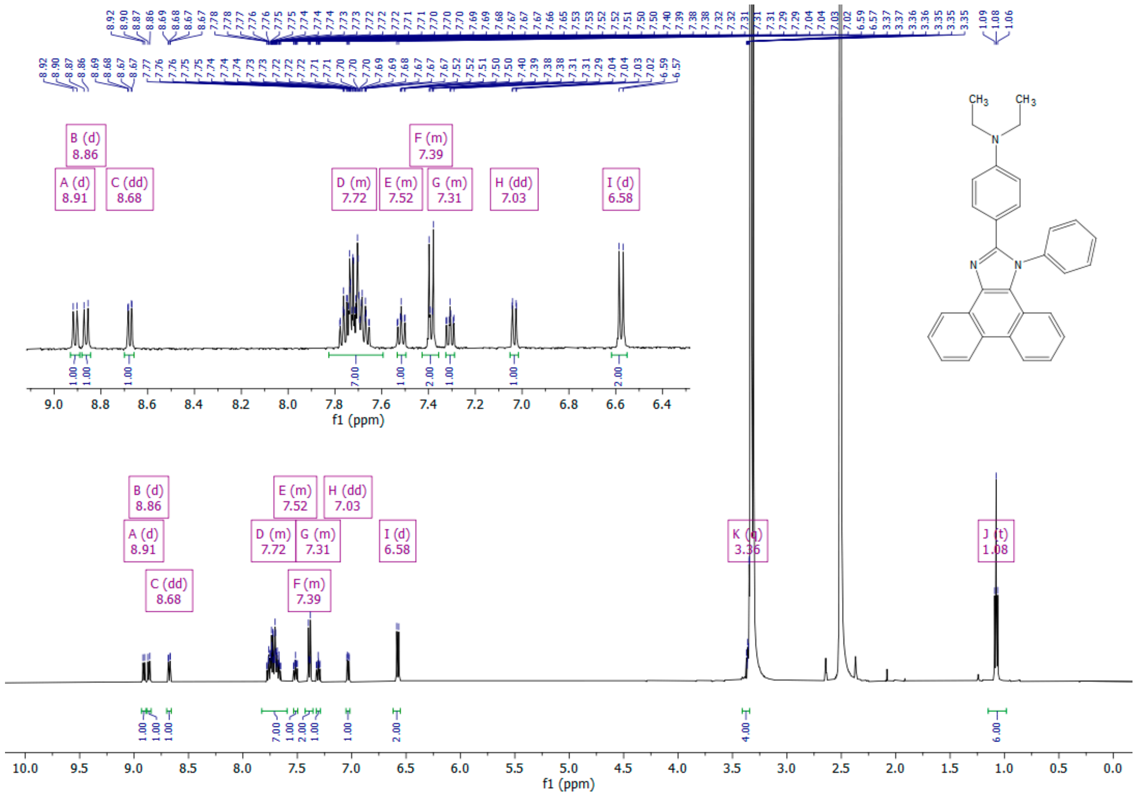

^{1}H NMR of AM-1

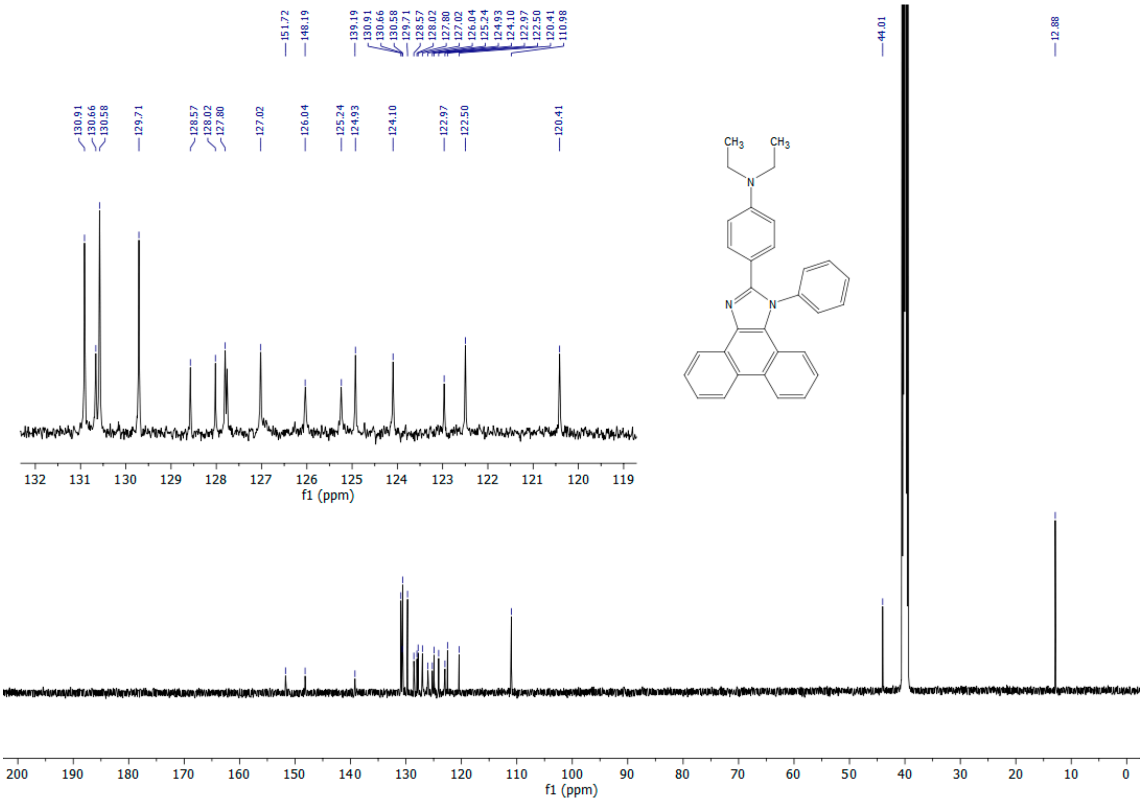

^{13}C NMR of AM-1

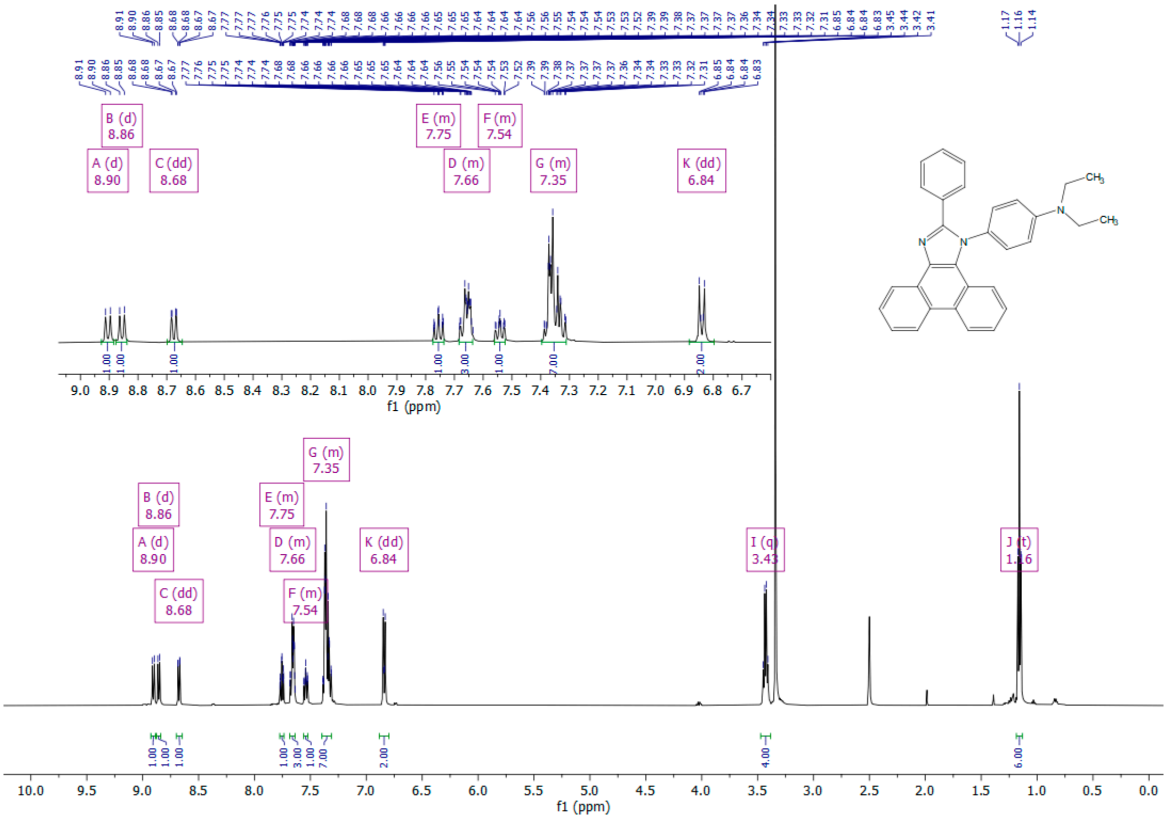

^{1}H NMR of AM-2

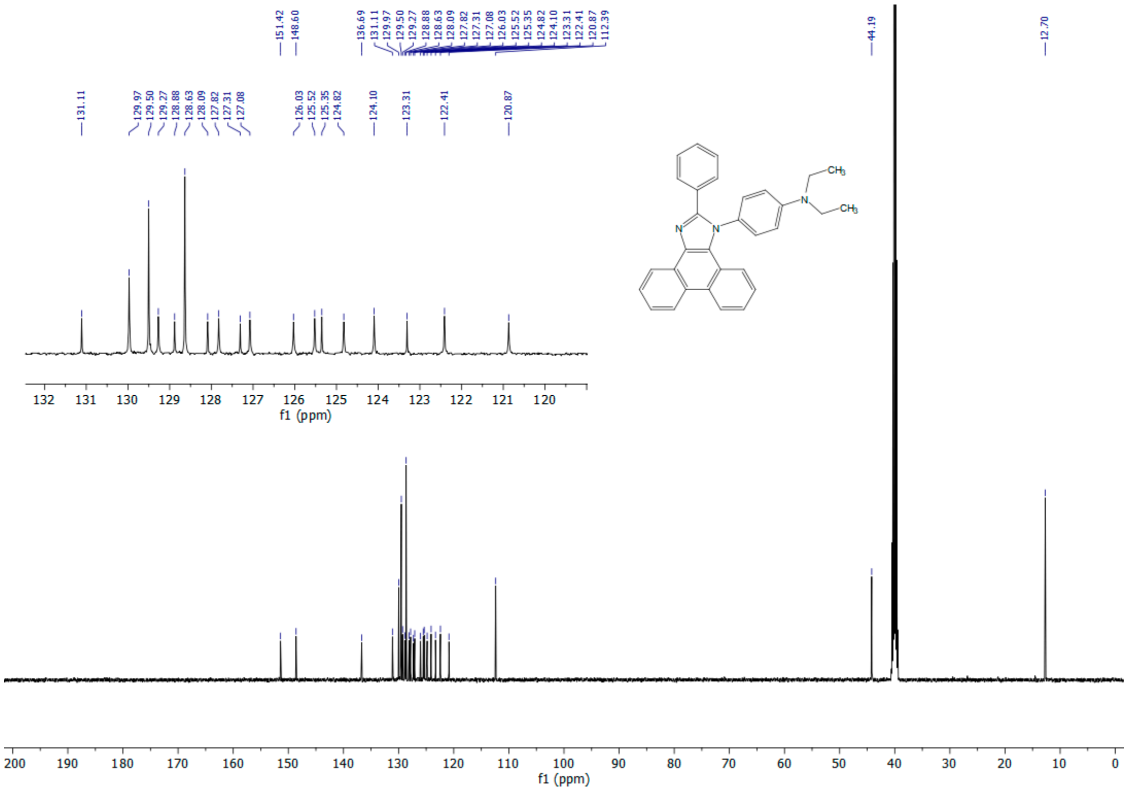

^{13}C NMR of AM-2

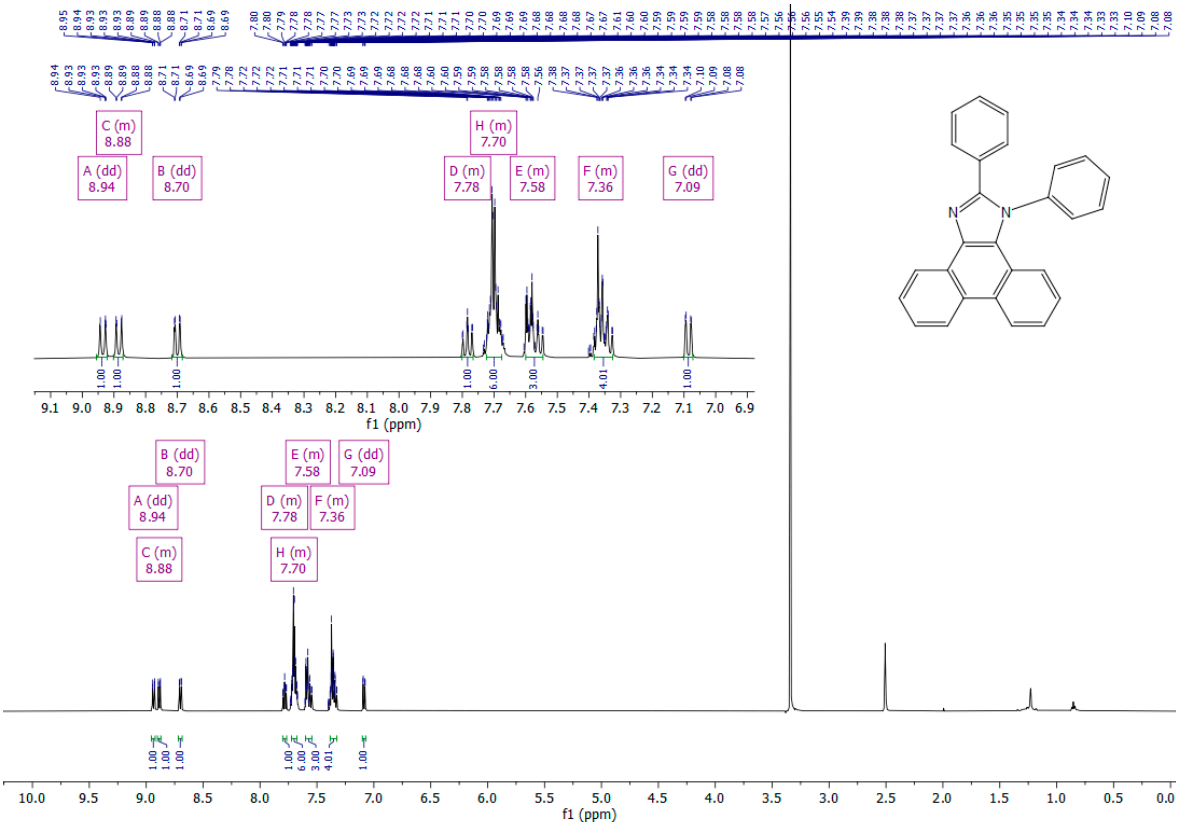

^{1}H NMR of AM-3

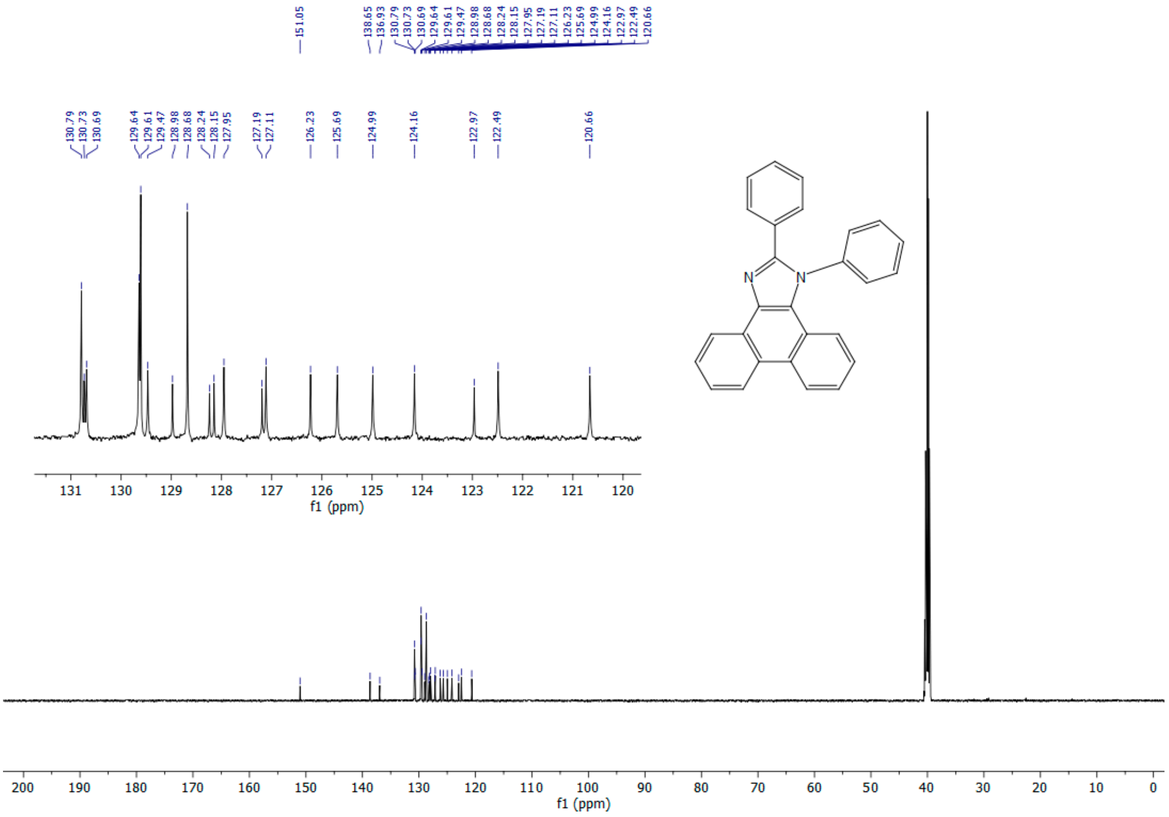

^{13}C NMR of AM-3

5. Thermal properties

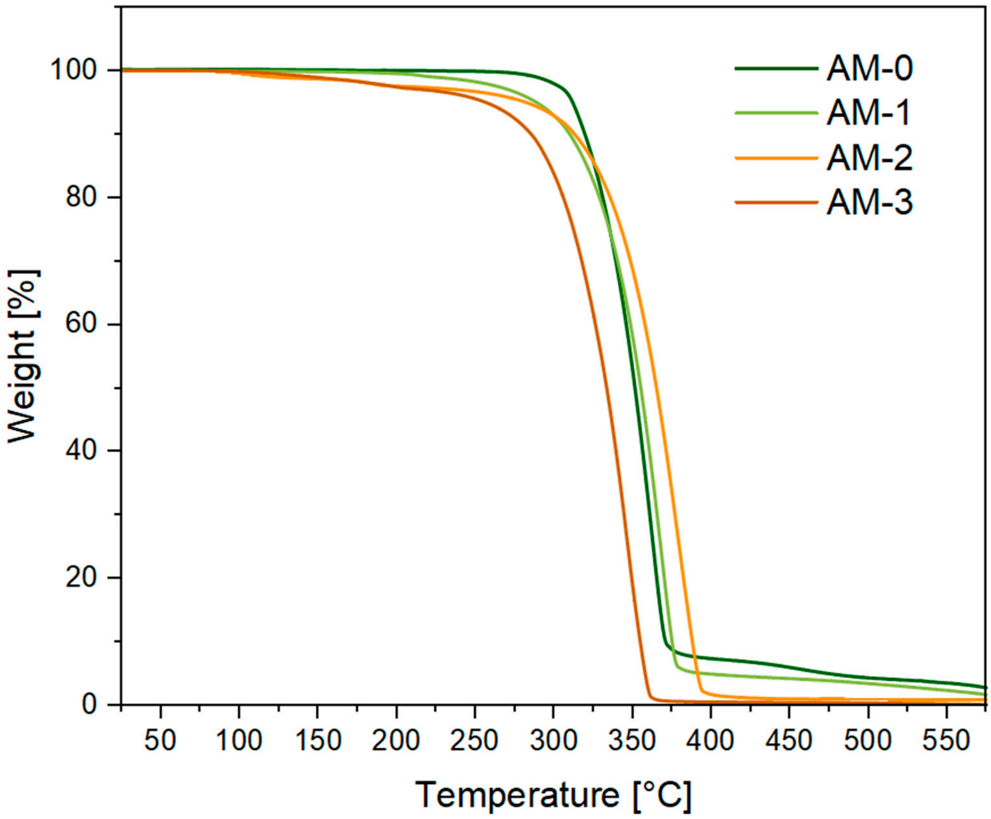

Figure S1. TGA thermogram – thermal properties of **AM-0 – AM-3** series.

6. Electrochemical properties

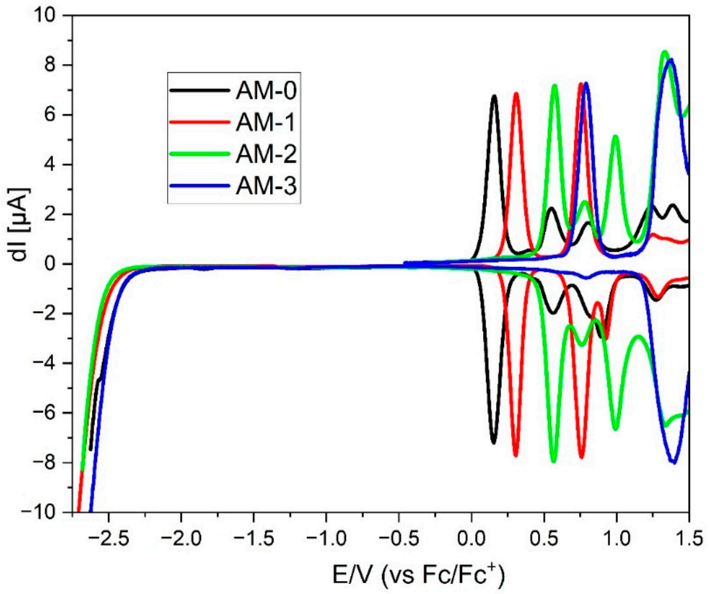

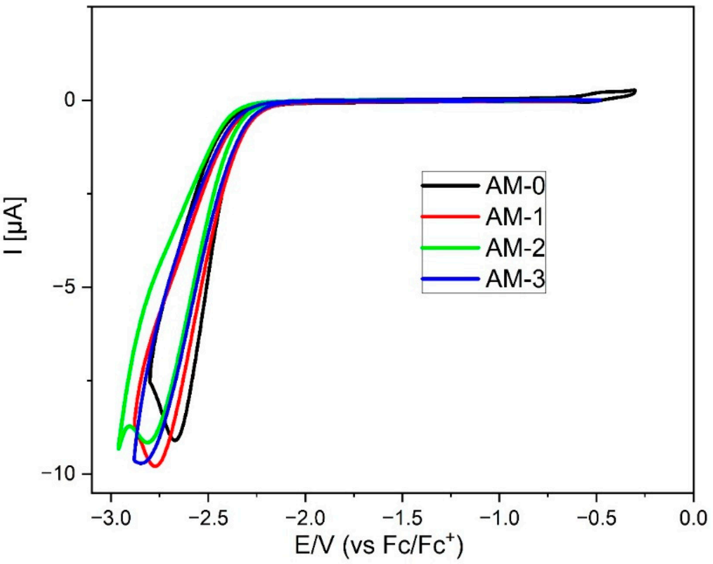

Figure S2. Differential pulse voltammetry and cyclic voltammograms of **AM-0 – AM-3** with sweep rate ν = 100 mV/s, 0.1 M Bu_4NPF_6 in CH_2Cl_2.

7. DFT calculation

	C2	N1
**AM-0**	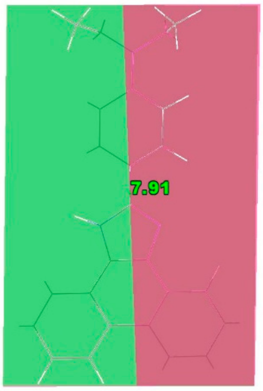	
**AM-1**	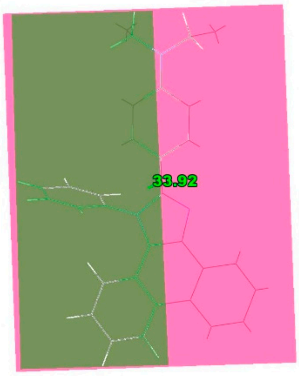	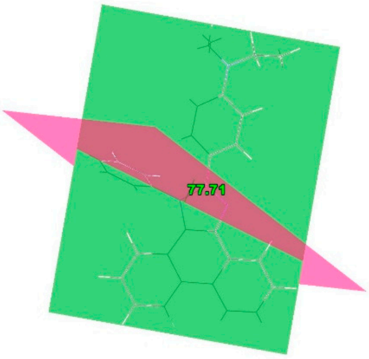
**AM-2**	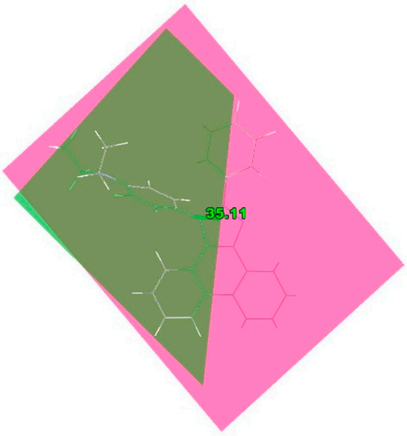	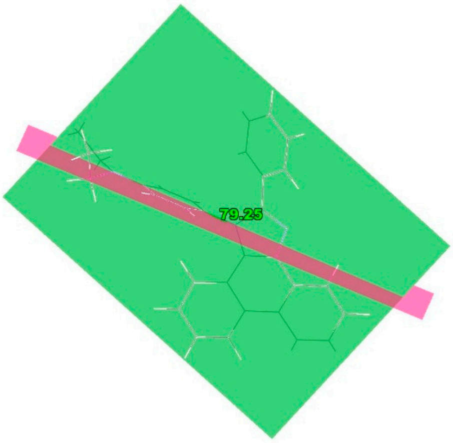

**AM-3**	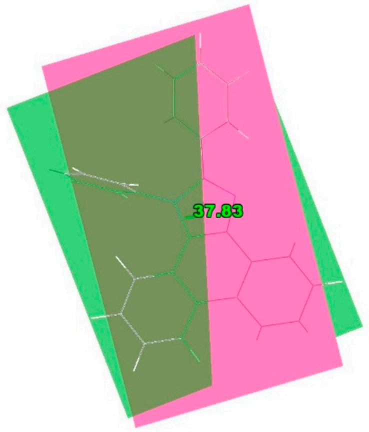	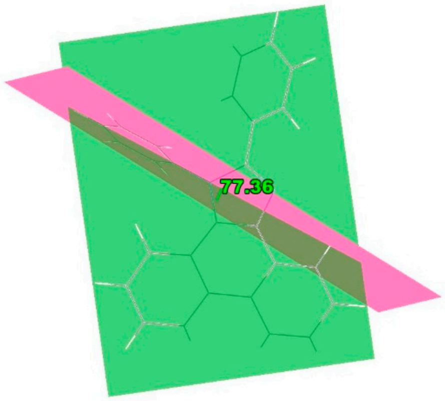

Figure S3. Dihedral angle between the C2 or N1 substituent and the central core of 1H-phenanthro[9,10-d]imidazole for **AM-0** – **AM-3.**

	HOMO	LUMO
**AM-0**	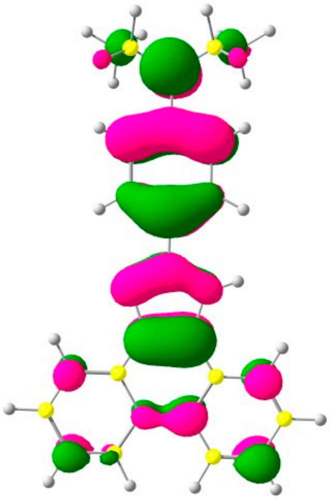	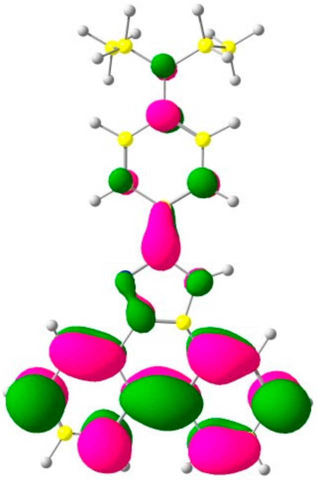
**AM-1**	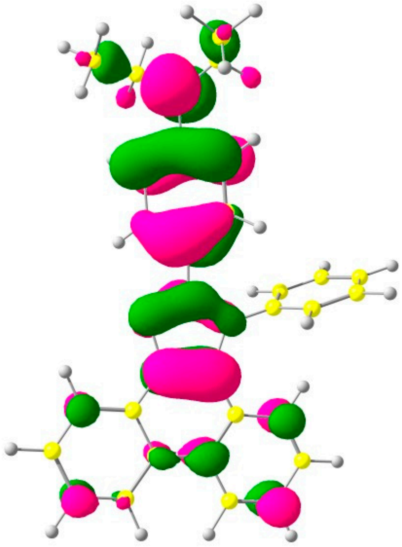	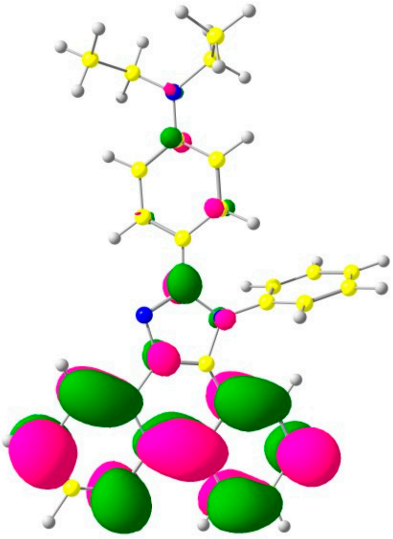
**AM-2**	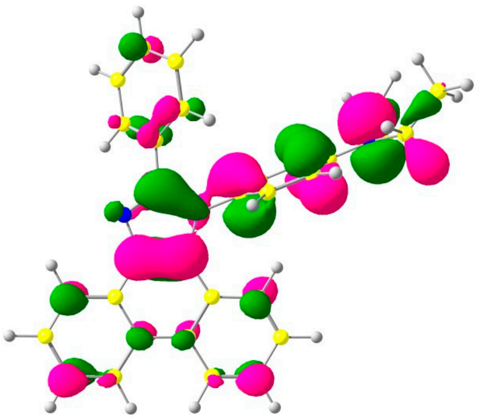	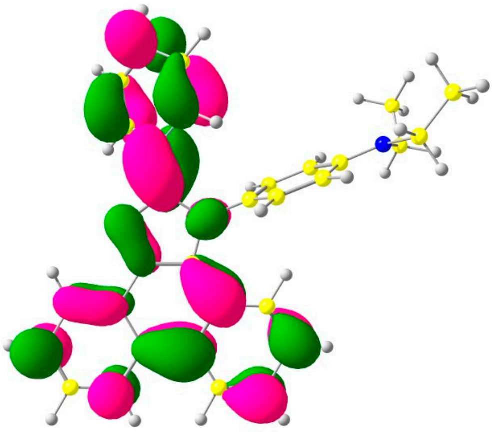

**AM-3**	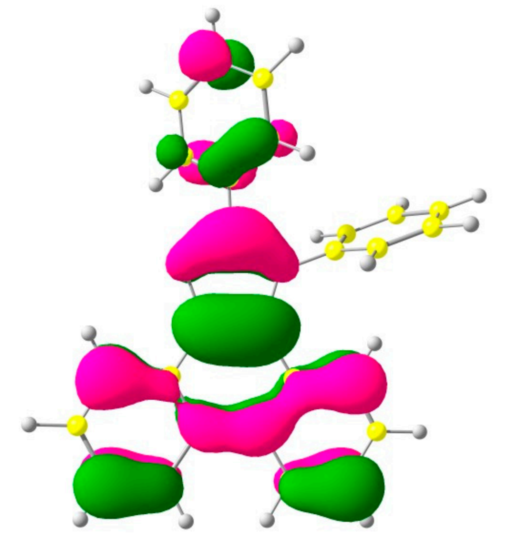	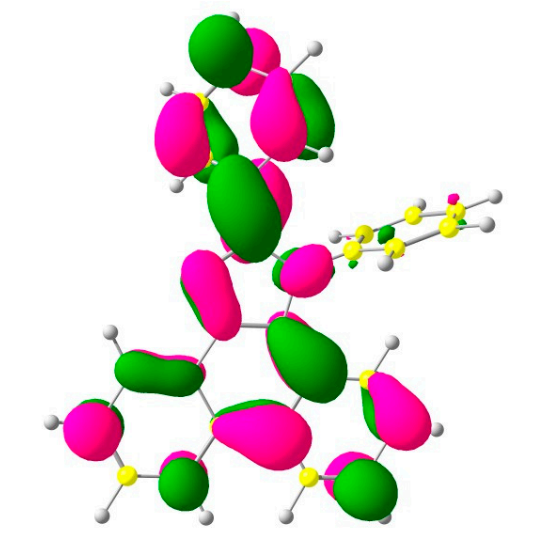

Figure S4. Contours of HOMO and LUMO orbitals for **AM-0** – **AM-3.**

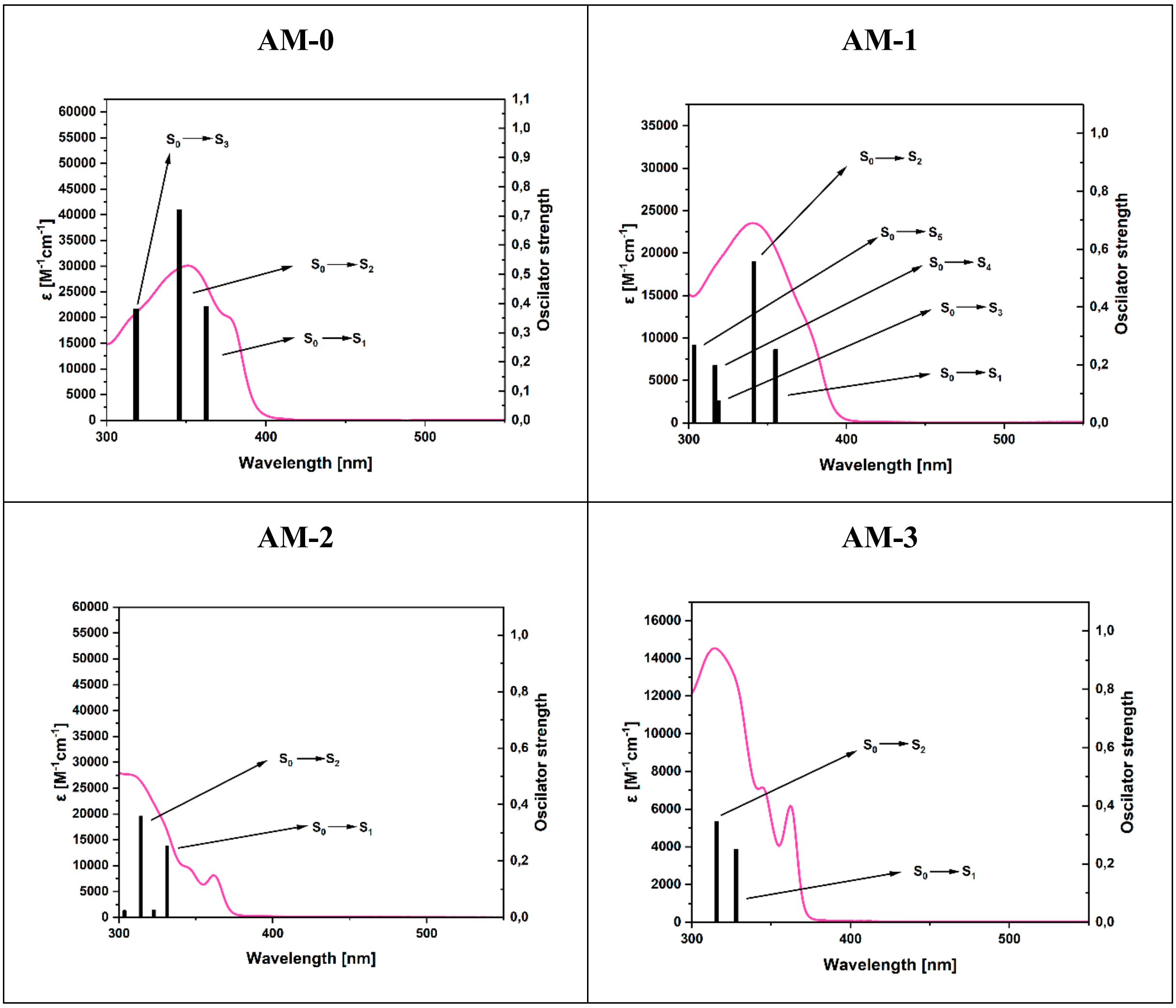

Figure S5. Experimental (pink line) absorption spectra and calculated transitions (black sticks) of **AM-0** – **AM-3** in dichloromethane.

Table S1. Electronic transition energies assigned to the lowest absorption bands of the **AM-0** – **AM-3** wavelengths.

Experimental absorption λ; nm (10^4 ε; M^{-1} cm^{-1})	Calculated transitions			
	Major contribution	E (eV)	λ (nm)	Oscillator strength
**AM-0**				
377.9 (19913)	$S_0 \rightarrow S_1$	3.42	362.6	0.3893
351.2 (30082)	$S_0 \rightarrow S_2$	3.59	345.7	0.7224
315.3 (20108)	$S_0 \rightarrow S_3$	3.89	318.6	0.3809
**AM-1**				
339.3 (23482)	$S_0 \rightarrow S_1$	3.49	355.0	0.2527
	$S_0 \rightarrow S_2$	3.63	341.2	0.5559
	$S_0 \rightarrow S_3$	3.89	318.8	0.0750
	$S_0 \rightarrow S_4$	3.92	316.6	0.1981
	$S_0 \rightarrow S_5$	4.08	303.4	0.2681
**AM-2**				
361.7 (8081)	$S_0 \rightarrow S_1$	3.74	333.4	0.2514
310.6 (27215)	$S_0 \rightarrow S_2$	3.94	314.3	0.3583
**AM-3**				
362.2 (6162)	$S_0 \rightarrow S_1$	3.78	328.0	0.2492
314.6 (14517)	$S_0 \rightarrow S_2$	3.93	315.7	0.3448

8. Optical properties

AM-0 – absorption normalized	AM-0 – emission normalized
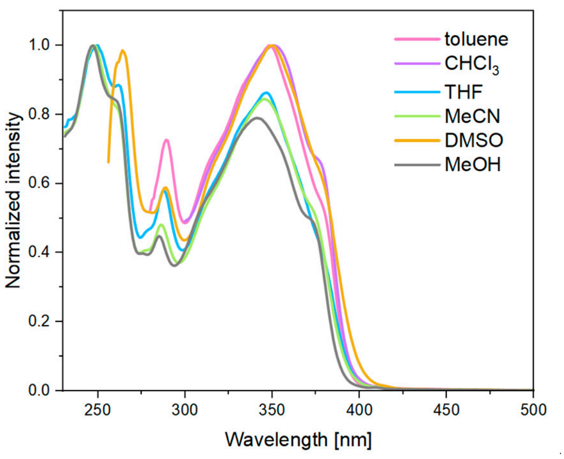	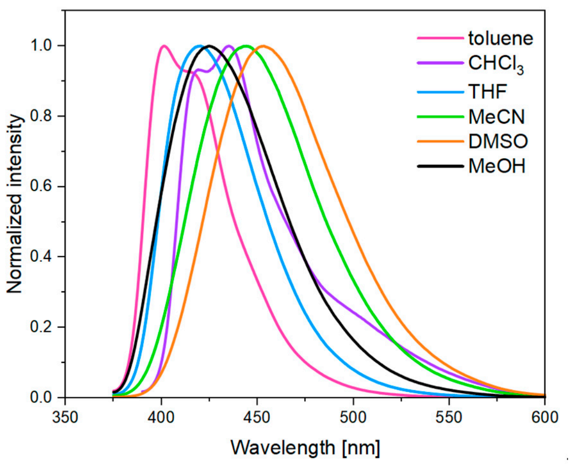
**AM-0 – absorption**	**AM-0 – emission**
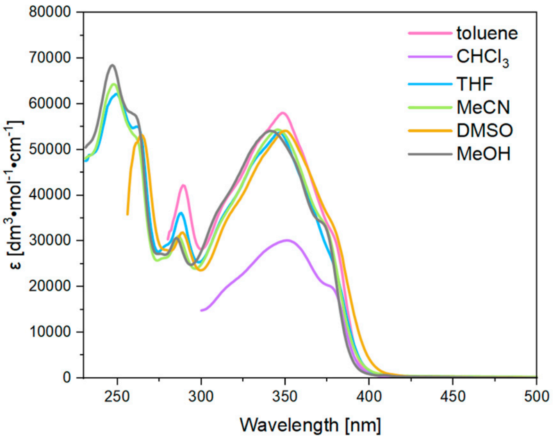	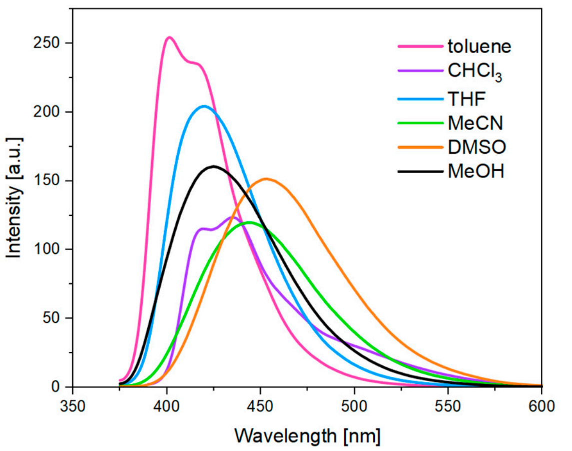
**AM-1 – absorption normalized**	**AM-1 – emission normalized**
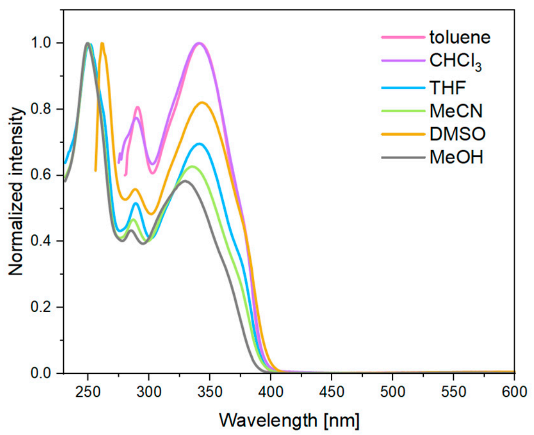	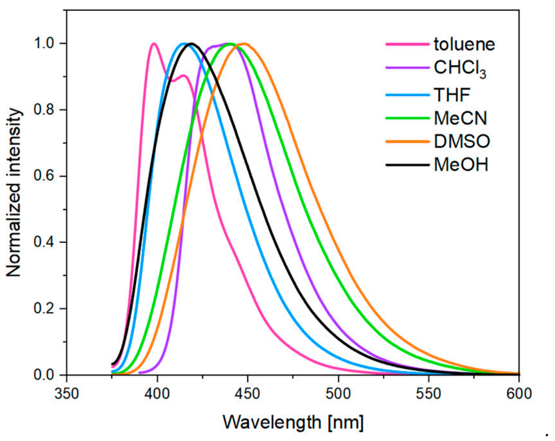

AM-1 – absorption

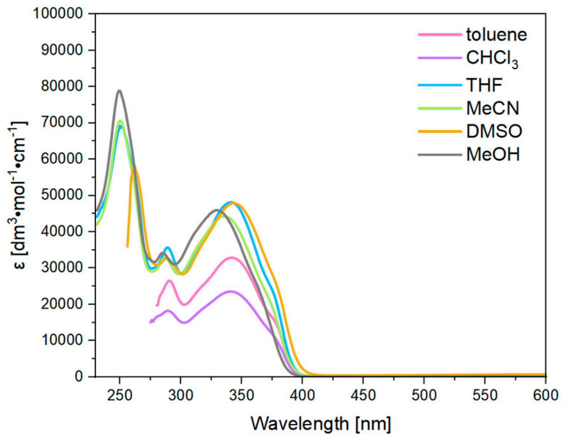

AM-1 – emission

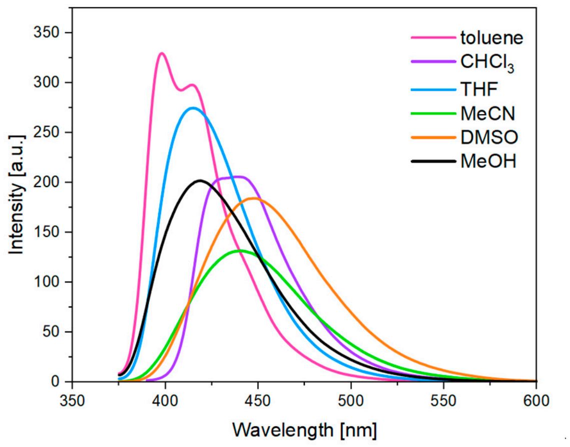

AM-2 – absorption normalized

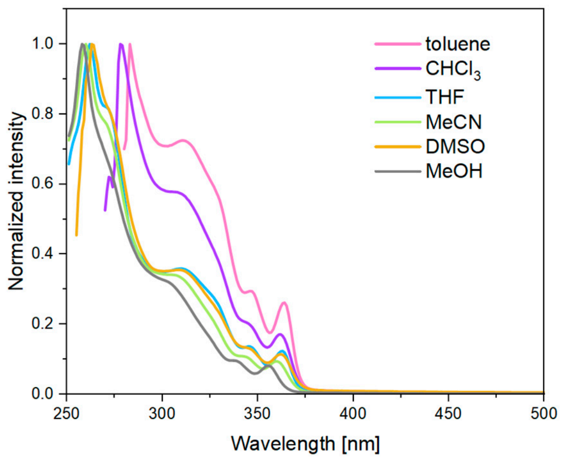

AM-2 – emission normalized

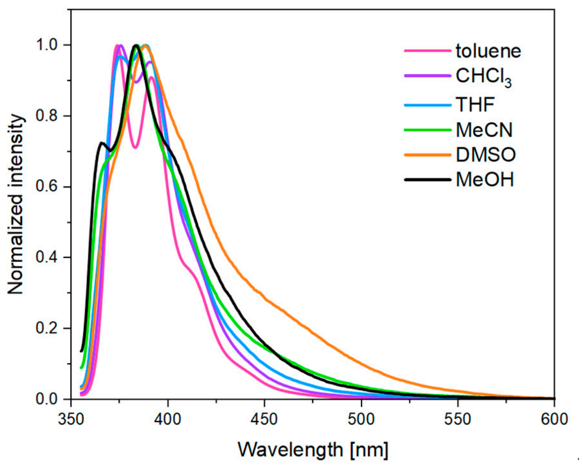

AM-2 – absorption

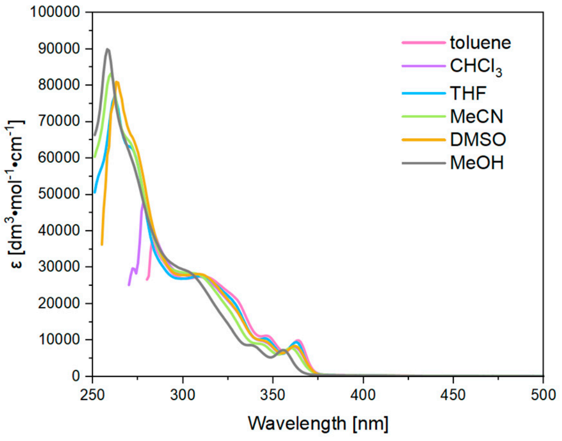

AM-2 – emission

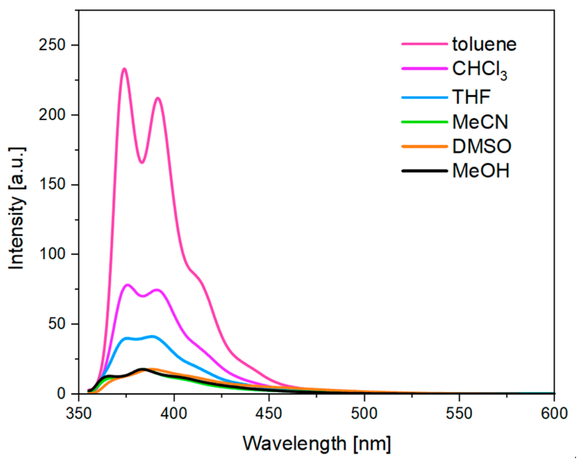

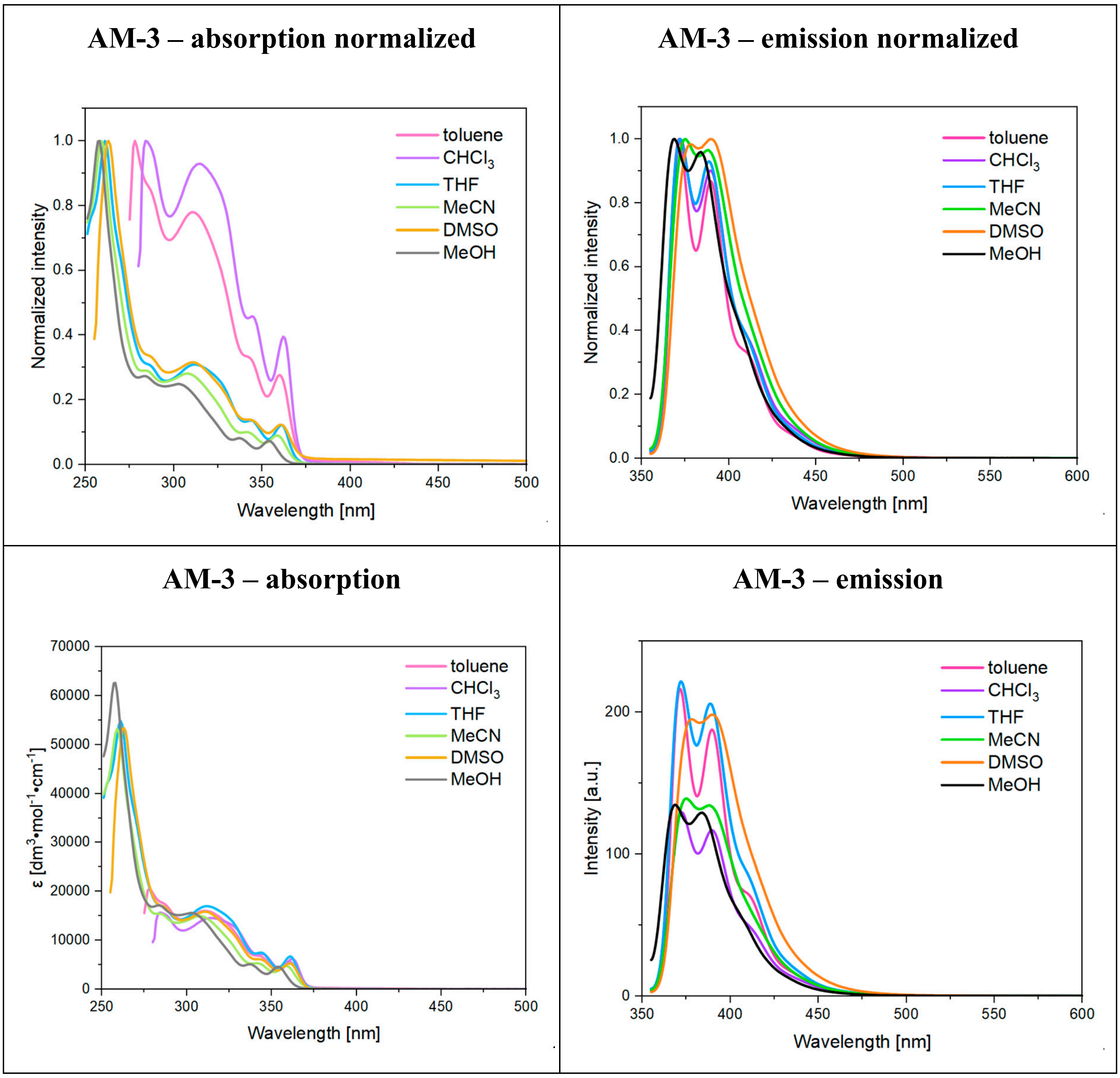

Figure S6. Absorption and emission spectra in different solvents for **AM-0** – **AM-3.**

Toluene – absorption

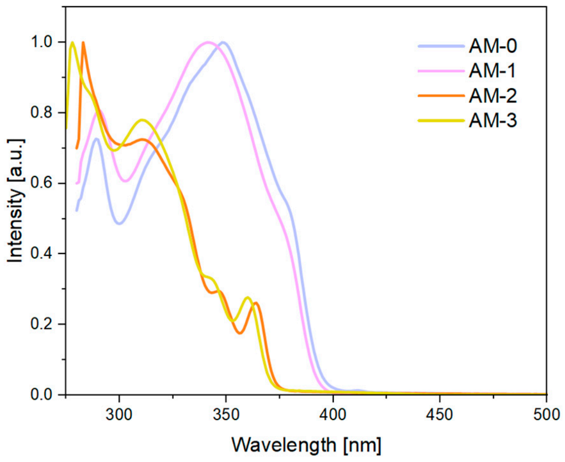

Toluene – emission

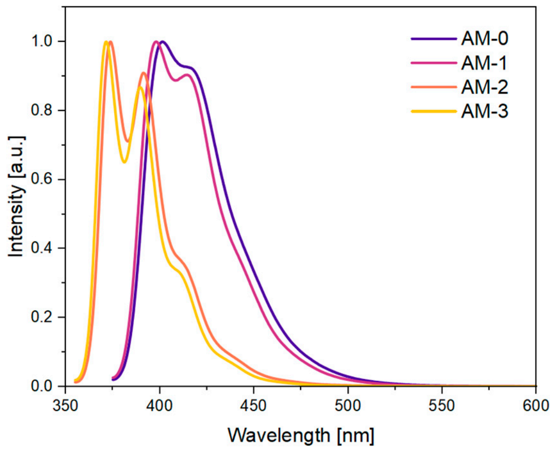

$CHCl_3$ – absorption

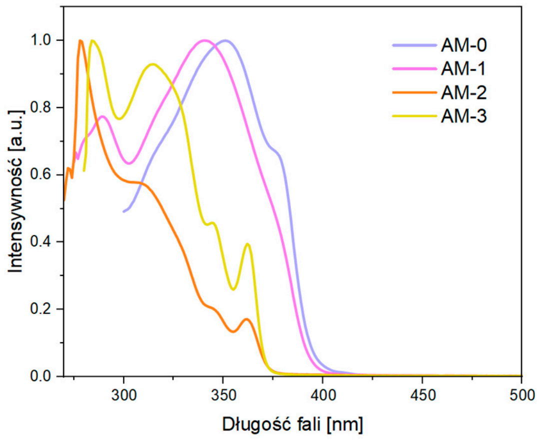

$CHCl_3$ – emission

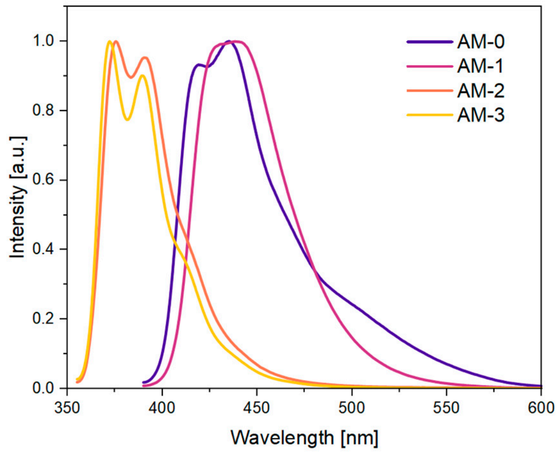

THF – absorption

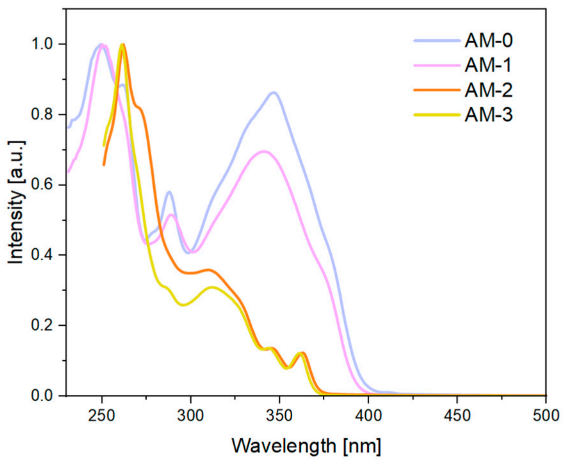

THF – emission

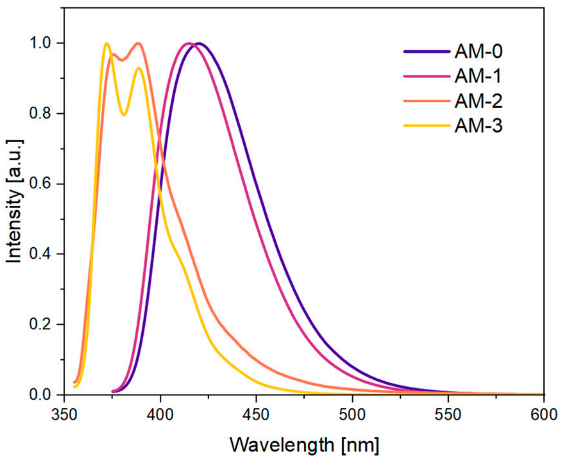

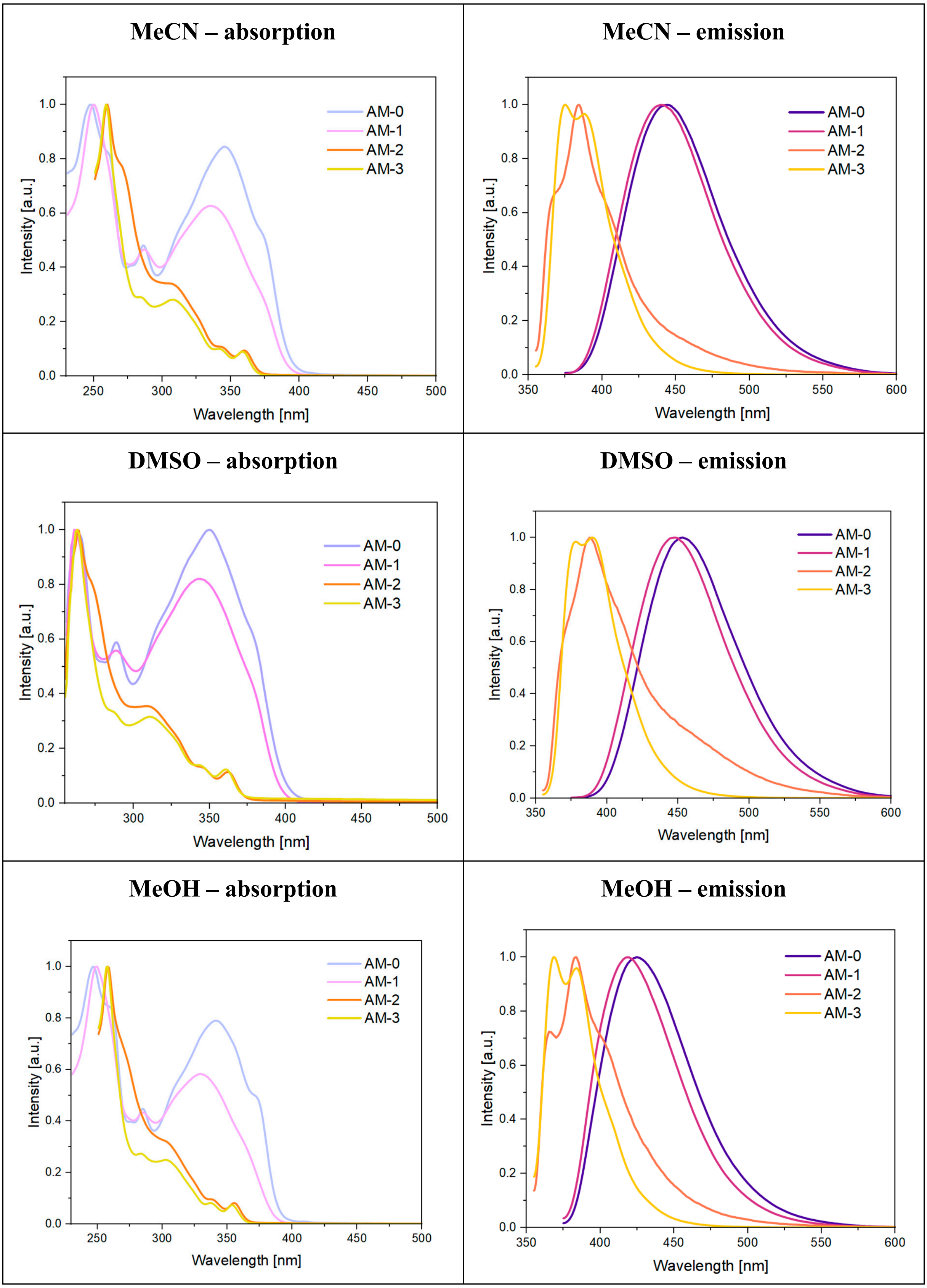

Figure S7. Comparison of absorption and emission spectra for **AM-0 – AM-3.**
